# Reconstructing visual illusory experiences from human brain activity

**DOI:** 10.1126/sciadv.adj3906

**Published:** 2023-11-15

**Authors:** Fan L. Cheng, Tomoyasu Horikawa, Kei Majima, Misato Tanaka, Mohamed Abdelhack, Shuntaro C. Aoki, Jin Hirano, Yukiyasu Kamitani

**Affiliations:** ^1^ Graduate School of Informatics, Kyoto University, Sakyo-ku, Kyoto 606-8501, Japan.; ^2^ ATR Computational Neuroscience Laboratories, Soraku, Kyoto 619-0288, Japan.

## Abstract

Visual illusions provide valuable insights into the brain’s interpretation of the world given sensory inputs. However, the precise manner in which brain activity translates into illusory experiences remains largely unknown. Here, we leverage a brain decoding technique combined with deep neural network (DNN) representations to reconstruct illusory percepts as images from brain activity. The reconstruction model was trained on natural images to establish a link between brain activity and perceptual features and then tested on two types of illusions: illusory lines and neon color spreading. Reconstructions revealed lines and colors consistent with illusory experiences, which varied across the source visual cortical areas. This framework offers a way to materialize subjective experiences, shedding light on the brain’s internal representations of the world.

## Teaser

Images generated from brain activity mirror illusory experiences, revealing neural representations behind subjective perception.

## INTRODUCTION

Visual illusions occur when perception of the world dissociates from sensory inputs. These illusions have been used to understand how the brain creates internal representations of the world. Physiological and neuroimaging studies have provided evidence of neural responses associated with illusory features. At the level of individual neurons in the visual cortex, some neurons exhibit similar responses to both actual and illusory attributes, suggesting the presence of a common neurobiological processing mechanism (*1*–*11*). On a broader scale, differential brain activity in some visual areas correlates with the perception of illusory attributes (*12*–*18*), and brain activity patterns can be classified according to differences in illusory experiences (*19*, *20*). However, despite these insights, the precise impact of these neural responses on overall perceptual experience remains elusive. Elucidating how the population activity of visual cortical areas translates into the exact content of an illusory experience is essential to fill a critical gap in our understanding of how brain activity represents perceptual experience.

We address this issue by reconstructing illusory percepts as images from brain activity at different levels of processing in the visual cortex. Recent decoding and reconstruction techniques have used deep neural network (DNN) representations translated from brain activity to enable the reconstruction of arbitrary stimulus images (*21*–*26*). These techniques have also facilitated the reconstruction of subjective content, such as mental imagery and attention-modulated perception, by using the same model that was trained on stimulus perception (*24*, *26*). Reconstruction provides a coherent representation of visual experience, encoded by neural population patterns and those mapped to DNN representations, which can be internally modulated to provide an alternative interpretation of sensory inputs. We hypothesize that an illusory stimulus would produce brain activity similar to that induced by a stimulus reflecting the subjective appearance of the illusion at specific stages of visual processing. This brain activity could be translated or decoded into DNN representations, which could then be converted into an image that exhibits the illusory attribute absent in the original stimulus.

## RESULTS

### Illusory stimuli and the reconstruction model

**Fig. 1.**
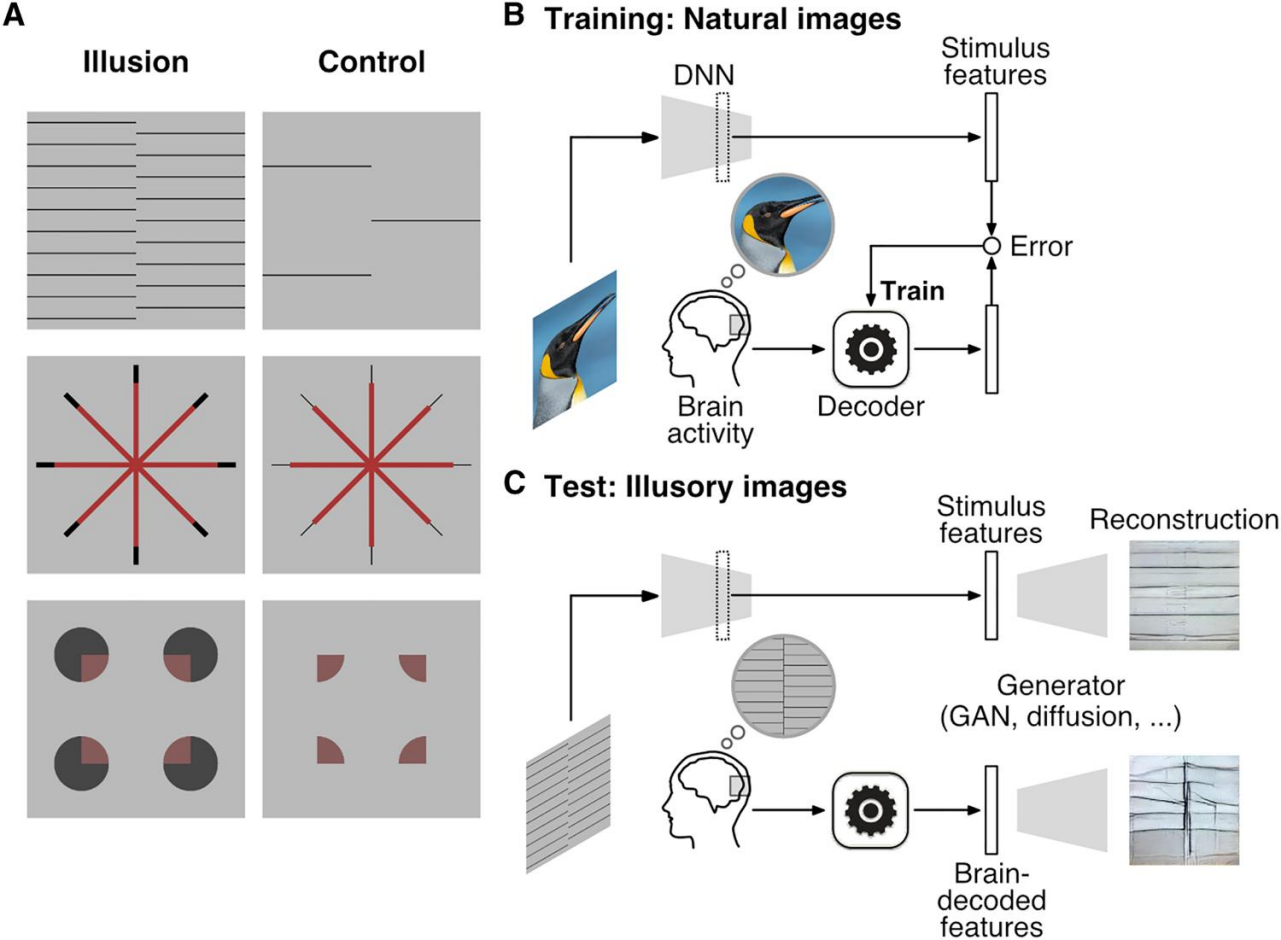
Illusory stimuli and image reconstruction procedure. (**A**) Example images of the illusion (left) and control (right) conditions: an illusory line induced by offset-gratings (top), the Ehrenstein (center), and Varin (bottom) configurations for neon color spreading. (**B**) Training. The stimulus features of natural images were extracted with a DNN pretrained for object recognition. Decoders were trained to predict the stimulus DNN features from fMRI responses to the same images. (**C**) Testing. Illusory images were presented together with control and positive control images. The stimulus features of a test stimulus or the DNN features decoded from fMRI responses to the test stimulus were passed to a pretrained generator for reconstruction.

We tested this idea using representative line and color illusions—illusory lines induced by offset-gratings and neon color spreading (Fig. 1A and fig. S1; see Materials and Methods). Illusory lines were produced by shifted line gratings (*27*, *28*). We used a total of six configurations of the inducer (0° and 90°) and illusory orientations (0°, 45°, 90°, and 135°). Neon color spreading is an illusion where the color spreads out of the stimulus region, producing the percept of a transparent color surface (*29*, *30*). We used two versions of neon color spreading: the Ehrenstein configuration (*31*, *32*), where the color is restricted to the line regions but appears to spread out to form a circular surface, and the Varin configuration (*33*), where the color is restricted to the wedge regions but appears to spread out to form a rectangular surface. For both illusions, we prepared control images that weakened or abolished the illusory percepts, and positive control images that mimicked the illusory percepts with real lines or a uniform color surface.

We adopted reconstruction models that consisted of DNN feature decoders and an image generator (Fig. 1, B and C). The DNN feature decoders were similar to those used in our previous studies (*22*, *24*). We used the unit activations of a feedforward convolutional neural network (*34*) as the target of decoding (Fig. 1B). The DNN feature decoders were trained on functional magnetic resonance imaging (fMRI) brain activity elicited by natural images of objects, material, and scenes including those added for this study (3200 images in total; see Materials and Methods). We used the fMRI signals of seven subjects from the visual cortex (VC), which covered both the early areas and the ventral object-responsive areas (see Materials and Methods). We assumed that most natural images would induce perceptions that closely mirror the physical features of the image (veridical perception) and that the trained decoders could adequately represent the mapping between brain activity and perceptual features, even without explicit information about subjective appearances.

At the test stage, we used the fMRI dataset measured for this study where the seven subjects were shown the illusory images as well as the control and positive control images in sequences interleaved with natural images (Fig. 1C; see Materials and Methods for exclusion criteria and missing data). Each test image was flashed at 0.625 Hz for 8 s in each trial and it was repeated across 20 trials. Using the trained DNN feature decoders (Fig. 1B), we obtained the decoded features from each single-trial brain activity of the test dataset (Fig. 1C). The decoded features were then input to a generator, which had been trained on a large natural image dataset to convert the stimulus DNN features of an image back to the original image. The stimulus DNN features of the test illusory images were also given to the generator to see if the involvement of brain activity is critical for the reconstruction of illusory features. We primarily used a generator based on a generative adversarial network (GAN) (*35*, *36*), which we had decided on before data collection. However, we also tested additional generators based on diffusion methods (*37*–*39*) and pixel optimization (*24*). While the three generators produced reconstructed images with different flavors, they yielded qualitatively similar results in terms of the visual features of interest. The chosen method was able to reconstruct natural images (fig. S2) with a quality that was on par with our previous study (*24*).

### Reconstructed images

**Fig. 2.**
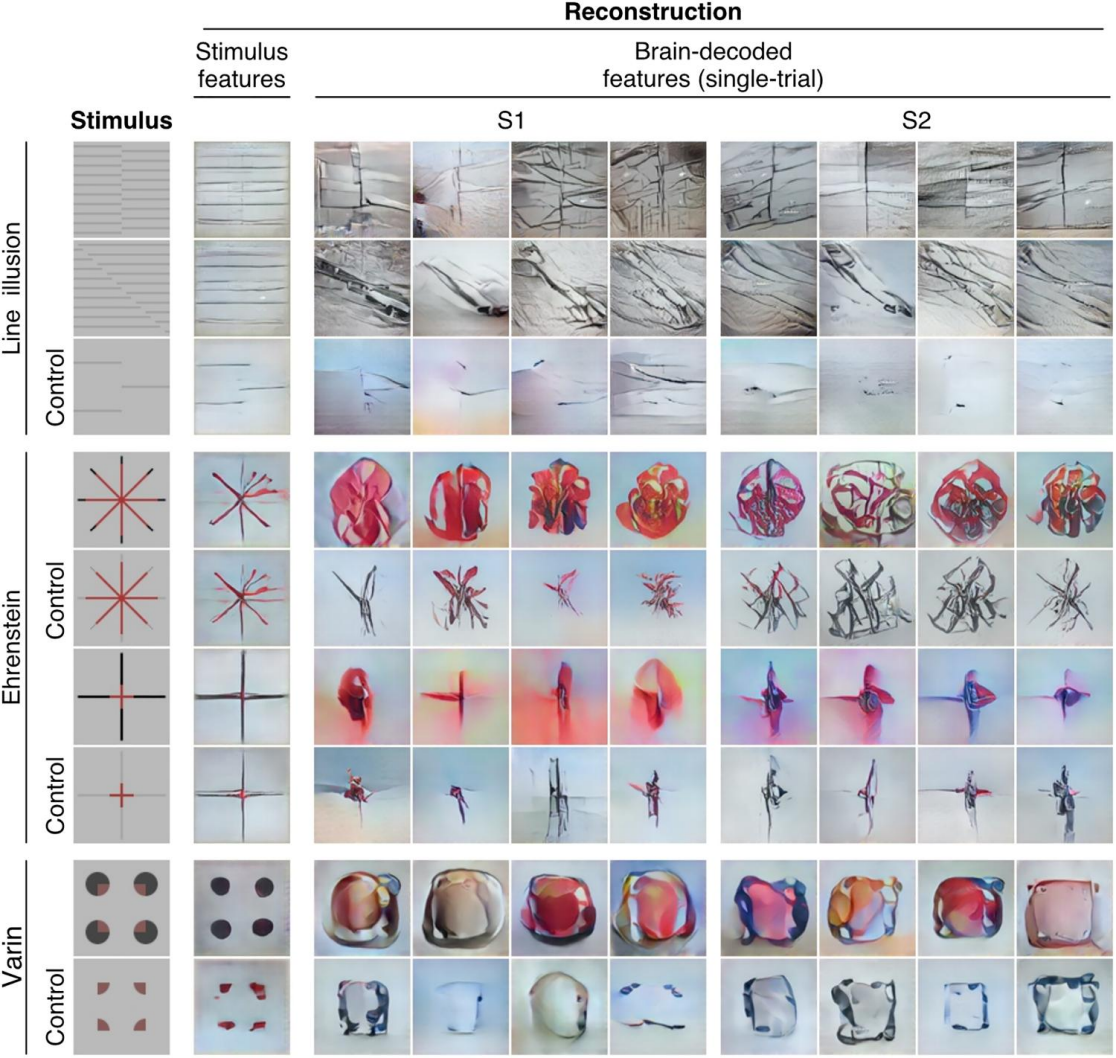
Reconstructions of illusory and control images. Reconstructions from stimulus features and from brain-decoded features are shown for two representative subjects (S1 and S2). Reconstructions from brain-decoded features were produced from single-trial (8-s) fMRI signals in the whole visual cortex (VC). Representative reconstructions from four different trials are shown for each subject.

We first confirmed that our reconstruction pipeline did not create spurious lines or colors congruent with the illusory percepts. We show reconstructions derived from stimulus DNN features for representative configurations (Fig. 2, “Stimulus features”). In all configurations, the reconstructions with stimulus features alone did not exhibit illusory components, even in the presence of noise (see fig. S3 for results from stimulus features plus the noise; see Materials and Methods). Although some DNN models can be trained to represent illusory appearances without the involvement of brain activity (*40*–*43*), the DNN model we used as the target for feature decoding is a feedforward convolutional neural network trained for object classification (*34*) and thus is unlikely to represent contextual features like illusory line and color. We found that individual units of the DNN representation did not show orientation or color tuning shared between real and illusory features (figs. S4 and S5). Thus, our reconstruction model itself seems to translate visual information represented in brain activity following the coding rules for veridical perception.

Building on these findings, we then examined the reconstructions with DNN features decoded from single-trial brain activity in the whole VC for each stimulus image (Fig. 2, right; two representative subjects and trials; see figs. S6 to S9 for results of others and fig. S10 for results of other generators). In the line illusions, the reconstructions contained line components of the illusory and the inducer orientations. The illusory orientation often appeared more prominent than the inducer orientation, and this effect was not limited to the specific image region where the illusory line was actually perceived. In the control condition, where there were fewer grating lines to weaken the illusory percept, the line components of the illusory orientation were not as prominent.

In the Ehrenstein configuration of neon color spreading, the reconstructions exhibited a more extensive colored region compared to the control, where a line-width gap was introduced to eliminate the illusory percept. For the Varin configuration, the control image was designed to suppress the color spreading but not the contour or shape component. The reconstructions from the illusory and control conditions showed a contour-like intensity profile, but color spreading was more pronounced in the illusory configuration. In both the Ehrenstein and Varin configurations, the outer inducer parts were often poorly reconstructed, likely due to the selective flashing of color regions primarily located in more central positions. The lower resolution of the peripheral representation may also contribute to the inferior reconstructions observed in the periphery.

### Quantitative analyses of illusory lines across multiple brain areas

**Fig. 3.**
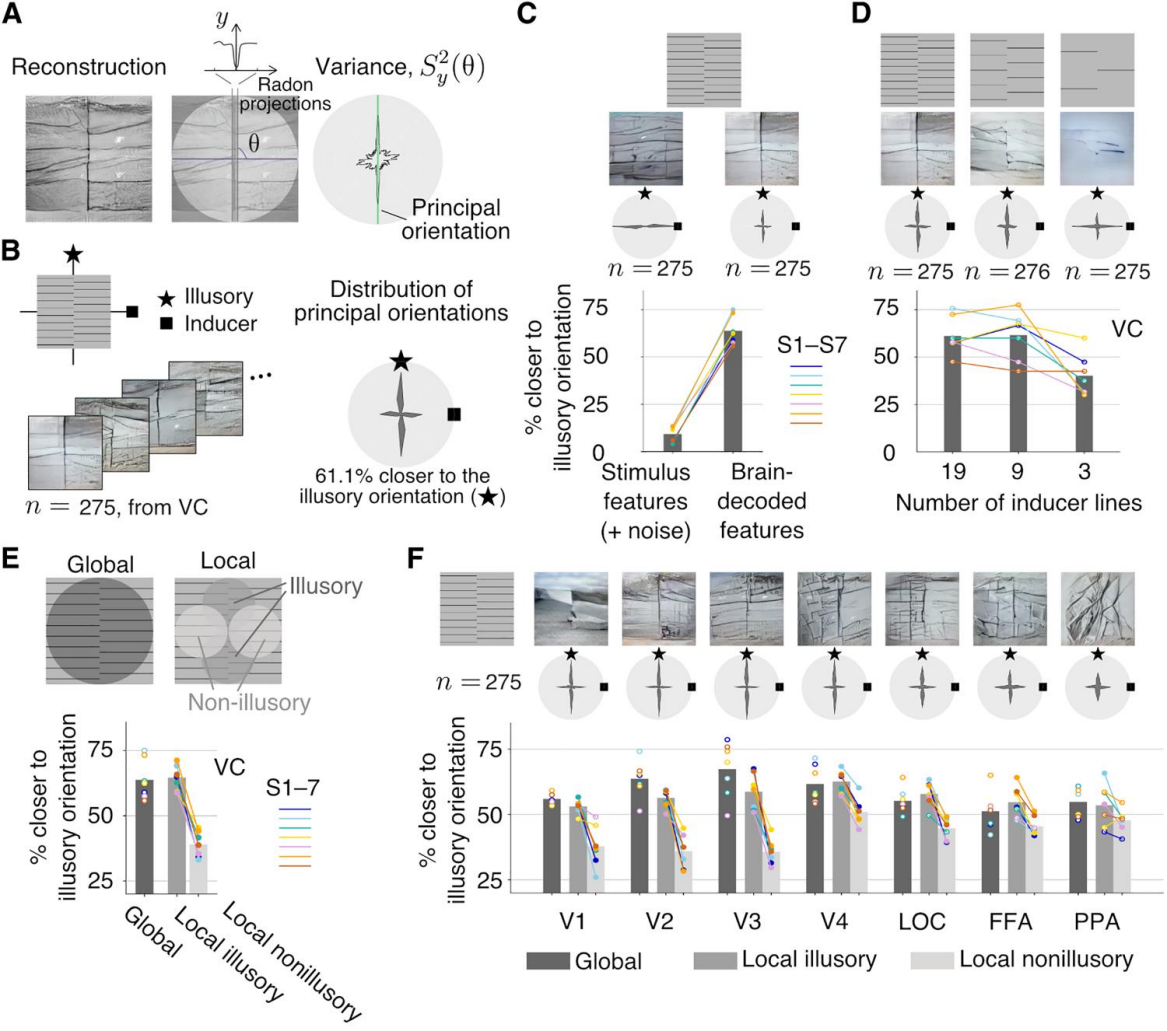
Evaluation of line illusion reconstructions. (**A**) Principal orientation detection. The orientation with the largest variance in Radon projections across line positions was identified as the principal orientation in an image. (**B**) Distribution of principal orientations in single-trial reconstructions from visual contex (VC) (results for seven subjects and all 90°-difference configurations are pooled, totaling *n* samples; bin size = 15°). An illusory orientation (star) and an inducer orientation (square) are shown for reference. (**C**) Comparison of reconstructions from brain-decoded features of VC and stimulus features with added noise. (**D**) Comparison of reconstructions with the different numbers of inducer lines. (**E**) Local presence of illusory orientation in reconstructions. (**F**) Comparison of reconstructions from individual visual areas. Reconstruction examples are from single-trial brain activity in VC [except in (F)] of subject S2. The polar plots show the distributions of the principal orientations pooled across all subjects and all 90°-difference configurations. The bar graphs indicate the proportions of principal orientations closer to the illusory than to the inducer orientation, pooled for all subjects and configurations. Color circles and lines indicate individual subjects. Comparisons with a statistically significant difference at the individual level are marked by solid circles [(C), (E), and (F)].

To quantify the reconstructed illusory lines (Fig. 3A), we detected the most apparent line orientation in each single-trial reconstruction using the Radon transform (*44*). We calculated Radon projections for line areas traversing the center of an image at each orientation. A prominent line would cause a substantial change in the projection value across parallel line areas at the line’s orientation. Thus, the orientation with the largest variance was defined as the principal orientation in each reconstruction (see Materials and Methods). The distribution of the principal orientations is shown in Fig. 3B by pooling single-trial reconstructions from VC for stimulus images with a 90° difference between the illusory and inducer orientations (*n* = 275 trials that survived the exclusion criteria from seven subjects; see fig. S11A for results of each subject). This distribution had a bimodal peak at the illusory and inducer orientations, with 61.1% of principal orientations closer to the illusory than the inducer orientation. The configurations of a 45° difference showed a similarly high closer-to-illusory proportion (65.2%; fig. S11, A and B).

Using these methods, we compared the reconstructions from brain-decoded features of VC with those from stimulus features (noise added to match the decoded features from each subject; see Materials and Methods). Consistent with representative reconstructions (Fig. 3C, top; see other reconstructions in figs. S3 and S6), the principal orientations with stimulus features were distributed mostly around the inducer orientation, unlike the bimodal distribution found with brain-decoded features (Fig. 3C, center; all 90°-difference configurations and subjects pooled). The closer-to-illusory proportion for stimulus features (plus noise) was near zero (Fig. 3C, bottom; all configurations pooled in each subject; one-sided *z* tests, *P* < 0.01 in seven of seven subjects). These results further confirm that the reconstructed illusory lines were derived from brain activity, not from the analysis pipeline itself.

In addition, we investigated the effect of the number of inducer lines. Prior research has shown that decreasing the number of inducer lines weakens illusory percepts (*28*). The reconstructions (Fig. 3D, top; additional examples in fig. S7) and distributions of the principal orientations (Fig. 3D, center; all 90°-difference configurations and subjects pooled; see results of each subject in fig. S12) indicate a gradual reduction in the strength of the illusory lines as the number of inducer lines is decreased. The closer-to-illusory proportion was similar at line numbers 19 and 9 and then decreased at line number 3 (Fig. 3D, bottom; all 90°-difference configurations and subjects pooled). Thus, the reconstructions appear to reflect the illusory appearance manipulated by the number of inducer lines.

The illusory line is perceived at the abutting portion of the inducer gratings. However, the method used above for detecting the principal orientation fails to capture this locality of the illusory percept. To examine the local presence of the illusory orientation in reconstructions, we analyzed local image regions and detected the principal orientation separately at (i) illusory regions where the illusory line was expected to be seen and at (ii) nonillusory regions where only inducer lines were expected to be seen (Fig. 3E, top). We calculated the closer-to-illusory proportions at each local region and the global region from the previous analysis (Fig. 3E, bottom; all configurations pooled in each subject). While the closer-to-illusory proportions for the local illusory regions were similar to those for the global region, the proportions for local nonillusory regions were significantly lower (one-sided *z* tests for proportions, *P* < 0.01 in seven of seven subjects; see fig. S11C for results of 90°- and 45°-difference configurations).

Reconstructions can be obtained using fMRI activity in individual visual areas [V1 to V4, lateral occipital complex (LOC), fusiform face area (FFA), parahippocampal place area (PPA); see Materials and Methods]. In Fig. 3F, we show examples of the reconstructions from individual areas (top; see fig. S13 for others), the distributions of principal orientations (center; pooled across 90°-difference configurations and subjects; see fig. S14 for results of 90° and 45° differences from each subject), and the closer-to-illusory proportions for the global, local illusory, and local nonillusory regions (bottom; all configurations pooled in each subject). Overall, V1 to V3 tended to show faithful reconstructions of illusory and inducer lines. V4 and the higher visual areas showed less localized illusory lines and poorly reconstructed inducer lines. The strength of the illusory orientation component for the global regions peaked around V2 to V4 (Fig. 3F, bottom). The difference between local illusory and nonillusory regions, which indicates the consistency with the local illusory percept, was large around V1 to V3 (one-sided *z* tests for proportions; *P* < 0.05 in five of seven subjects at V1; seven of seven at V2, V3, and V4; six of seven at LOC; four of seven at FFA; and three of seven at PPA). The overall global and local trends in reconstructions of illusory images were found to mirror the trends seen for the positive control images, though the actual strength of the illusory line was weaker than that of the real line in V1 to V3 (fig. S15). The strength of illusory orientation components (indicated by global closer-to-illusory proportions) in V1 to V4 and LOC seems to reflect the strength of subjective line perception manipulated by the number of lines (fig. S16). The results suggest that the local representations of illusory lines are formed in early visual areas and that illusory and real lines are similarly represented across visual areas.

### Quantitative analyses of illusory color across multiple brain areas

**Fig. 4.**
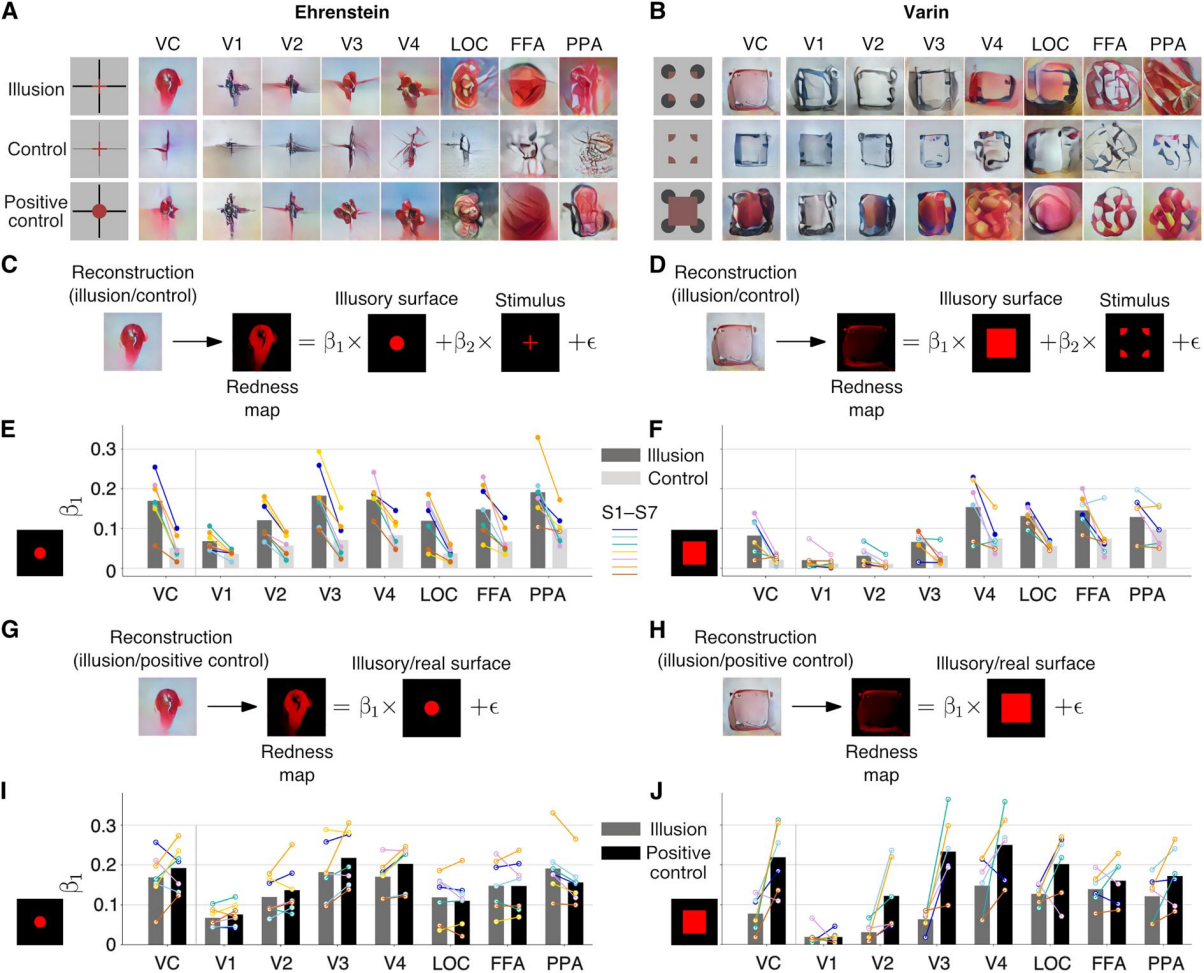
Evaluation of neon color spreading reconstructions. (**A** and **B**) Representative single-trial reconstructions of the illusion (top), control (center), and positive control (bottom) conditions for Ehrenstein from subject S1 (A) and for Varin from subject S2 (B). (**C** and **D**) Illustration of regression analysis for comparing the illusion and control conditions for Ehrenstein (C) and Varin (D). The redness map of a reconstructed image was fitted by those of the illusory surface (expected region of color filling-in) and the stimulus. (**E** and **F**) Comparison of the illusory surface coefficient values between illusion and control conditions for Ehrenstein (E) and Varin (F). Results for all configurations (sizes and numbers of lines) and seven subjects are pooled for Ehrenstein. Results for six subjects are pooled for Varin. Color lines indicate the results of individual subjects. Comparisons with a statistically significant difference at the individual level are marked by solid circles. (**G** and **H**) Illustration of regression analysis for comparing the illusion and the positive control conditions for Ehrenstein (G) and Varin (H). The redness map of a reconstructed image was fitted by that of the illusory or real surface. (**I** and **J**) Comparison of the illusory surface coefficient values between the illusion and the positive control conditions for Ehrenstein (I) and Varin (J), pooled as in (E) and (F).

We next produced reconstructions for neon color spreading from individual visual areas and VC (Fig. 4, A and B; see figs. S8, S9, S17, and S18 for additional examples). Reconstructions from the illusion condition of the Ehrenstein configuration exhibited red regions across areas, similar to the positive control condition, with lower areas more accurately depicting the sizes of red regions and inducer lines (Fig. 4A). However, in the control condition, where a gap in line width was introduced to abolish or weaken illusory color spreading, the color was largely absent in the reconstructions, even though the illusion and control stimuli included the same red regions. Reconstructions from the Varin illusion condition exhibited similarity to the positive control condition only in mid-to-higher areas, showing broad red regions (Fig. 4B). The color was barely present at V1 to V3, while faithful reconstructions of the real color could be produced from V2 and V3 in the positive control condition. The inducer regions were poorly reconstructed, even in lower areas, in the illusion and the positive control conditions. Although square-like outlines were seen in the reconstructions from the lower areas, they did not coincide with the illusory square shape but seemed to enclose the entire stimulus region. In the control condition, the color was almost absent: Square outlines were reconstructed more clearly from the lower areas and appeared to align the illusory square without color spreading.

To quantify color spreading, we performed regression analysis on the pixel color values in each reconstructed image (Fig. 4, C and D; see Materials and Methods). We created the redness (saturation) maps from the original red-green-blue (RGB) values of reconstructed and stimulus images. In addition, we prepared the redness maps for the expected illusory surface regions. The profile of the redness map from a reconstruction was fitted by those of the expected illusory surface region and the stimulus. The illusory surface regressor was shared between the illusion and the corresponding control conditions for comparison. The illusory surface coefficient (β_1_) was used as the measure for illusory color reconstruction. Regression coefficients were calculated for all individual trials (reconstructions) and pooled across different configurations (sizes and numbers of lines for Ehrenstein) in each subject and brain area.

For Ehrenstein, the illusory surface coefficient was generally higher in the illusion condition than in the control condition (Fig. 4E; one-sided *t* tests in individual subjects; *P* < 0.05 in seven of seven subjects at VC; five of seven at V1; seven of seven at V2, V3, V4, LOC, and FFA; and six of seven at PPA; see fig. S19A for results with different stimulus configurations). V2 to V4 and higher areas appear to show robust illusion effects (see fig. S20 for results with individual trials in each subject). For Varin, the illusory surface coefficient was greater in the illusion than in the control condition in mid-to-higher areas, but the effects in individual subjects were less robust than those with Ehrenstein (Fig. 4F; one-sided *t* tests in individual subjects; *P* < 0.05 in three of six subjects at VC; two of six at V1, V2, and V3; three of six at V4; four of six at LOC; three of six at FFA; and one of six at PPA; see fig. S21 for results with individual trials in each subject).

Similarly, we performed regression analysis in which the profile of the redness map from a reconstruction was fitted by the illusory or real color surface for the illusion and the positive control conditions (Fig. 4, G and H). Whereas surface coefficients were comparable between the illusion and the positive control conditions across brain areas for Ehrenstein, lower surface coefficients were observed in the illusion compared to the positive control condition in low-to-mid areas for Varin (Fig. 4, I and J; see figs. S22 and S23 for results with individual trials in each subject). In addition, large-sized Ehrenstein configurations tended to show lower illusory surface coefficients for the illusion than the positive control condition in the low-to-mid brain areas (fig. S19B). Thus, the strength of illusory color reconstruction across brain areas may depend on the spatial extent of filling-in and the stimulus configurations.

## DISCUSSION

We have demonstrated the reconstruction of illusory percepts as images from single-trial brain activity, using the computational model that learns the coding scheme linking non–illusory stimulus–induced perception and brain activity. The reconstructions obtained from the VC resembled the illusory percepts, to the point where the relevant attributes were perceptually recognizable. It is important to note that neither the stimulus features of the illusion-inducing stimuli nor the brain-decoded features of the control images produced such reconstructions. One notable advantage of our approach is the ability to externalize mental contents, going beyond the conventional method of testing qualitative hypotheses. By representing mental contents in a manner comprehensible to others, we offer a means of sharing and understanding subjective experiences. However, the current methods have limitations including the lack of spatial resolution, in particular for the peripheral vision, resulting in a poor reconstruction of the inducers. In addition, the reconstructed images are distorted presumably due to inherent biases in the reconstruction model.

We used models trained on natural images to decode illusory percepts formed from artificial shapes. The ability to generalize to such out-of-sample images is crucial for such models. This may be achieved in part by the latent feature dimensions of the DNN that effectively capture image-level information while being compatible with the representations in the brain (*24*, *45*). Furthermore, decoders should be trained on a diverse set of images where the features of individual dimensions appear with sufficient frequency (*21*, *46*). While it is not necessary for the images to be of the same category between the training and test stages, it is important to maintain commonalities at the level of the feature dimensions. The choice of a generator is also important in this context. Even though the generators were trained on identical image sets, the diffusion generator exhibited image components that resembled natural objects, such as flowers, while the GAN generator did not (see Fig. 2 and fig. S10A). Recent findings suggest that while both GAN and diffusion models tend to memorize training images, diffusion models recall more data than GANs, replicating the pixel-level details, structures, and styles of the training images (*47*, *48*). Techniques to enhance the generalization capabilities of generators are currently in development (*49*).

The reconstructions from individual areas unveiled the strength of illusory representation and the extent to which it is shared with real stimuli at different processing stages. Specifically, regarding illusory lines, our results align with previous findings (*1*–*5*, *7*–*10*, *13*), showing the involvement of low-level areas and LOC in processing both illusory and real lines. Our reconstructions further revealed that local illusory lines, which accurately reflected perceptual experiences, were better represented in areas V1 to V3, while central illusory lines tended to dominate in higher areas. With regard to illusory color, previous research has produced mixed findings concerning its representation in the low and middle areas (*12*, *14*, *19*, *20*). Our results, however, show the participation of multiple areas in representing illusory color, extending even to higher areas within the ventral cortex. Previous studies often categorized widespread brain activity using broad labels, which could be confounded by other factors, such as the properties of inducers. This challenge becomes more pronounced in higher brain regions, where expansive receptive fields encompass both illusory and inducer components. In contrast, our approach may offer a more precise mapping between visual features and brain activity by reconstructing spacial configurations in an image.

Our findings suggest that neural representations of illusory percepts vary not only between different visual attributes such as line and color, but also among configurations for the same attributes. Specifically, for the Ehrenstein configuration of neon color spreading, we observed that illusory color representations spanned from low to high areas, with overlaps with real color representations. In contrast, in the Varin configuration, we found illusory color representations primarily in mid-to-high areas, whereas early areas manifested a more pronounced representation of real color as compared to illusory color. Computational models attribute neon color spreading and related phenomena to mechanisms that involve lateral interactions and top-down feedbacks (*30*, *41*, *50*–*52*). While the Ehrenstein and Varin configurations might employ the same mechanisms, the distinct spatial arrangement of their inducers could lead to varying intensities of lateral and top-down interactions. For example, in the Varin configuration, the red wedges are situated in the peripheral areas, which could hinder color diffusion from the periphery to central vision through lateral connections. Concurrently, the top-down feedback from higher brain regions may not be sufficiently strong to activate neurons in the lower areas.

Illusions serve as tools to investigate the neural underpinnings of consciousness by distinguishing brain activity associated with subjective experience from that linked to the stimulus itself. Although various theories suggest different brain regions and mechanisms as the underpinnings of consciousness (*53*–*59*), our findings highlight the difficulty in identifying a singular brain region that consistently reconstructs illusory experiences across all conditions. Thus, multiple neural mechanisms across various areas are likely implicated in the processing and interpretation of illusory stimuli. This nuanced understanding provides valuable insights into the neural representation of conscious experience, imposing constraints on theories of consciousness. However, it is important to acknowledge that our method cannot establish a causal relationship between brain activity and subjective percepts in the way that lesion studies can. Besides, our methods primarily illuminate representations rather than processing or dynamics. To further our understanding, high-resolution and multimodal brain measurements and manipulations would serve as beneficial supplementary tools.

In conclusion, our reconstruction approach effectively bridges the gap between internal representations and their external manifestations. This method paves the way for further explorations of, and communications about, the internal representations of the perceptual world that reside within the brain.

## MATERIALS AND METHODS

### Subjects

We collected fMRI data from seven healthy subjects, four males and three females (aged 25 to 36 years). All subjects provided informed consent before the experiment, with the study protocol having been reviewed and approved by the Ethics Committee of the Graduate School of Informatics at Kyoto University (approval no.: KUIS-EAR-2017-002). The subjects had normal or corrected-to-normal vision. Four subjects (subjects S1 to S4) were the same as those in previous studies (*24*, *26*). Therefore, we used their published data (training session; available from https://openneuro.org/datasets/ds003430/versions/1.1.1 for subjects S1 to S3 and https://openneuro.org/datasets/ds001506/versions/1.3.1 for subject S4) as a subset of training data and collected additional training data. For subjects S5 to S7, training datasets were newly acquired with fewer repetitions in order to conserve resources (“fMRI Experiments: Training session”). For subjects S5 and S7, the same image set as subjects S1 to S4 was used, while for subject S6, 32 training images were replaced due to unpleasant object categories for the subject. Test datasets were newly acquired for all subjects though subject S4 lacked a few sessions of the test dataset (“fMRI Experiments: Test session”).

### Visual stimuli

#### Natural images

A total of 3200 natural images were downloaded from the online image databases. We obtained 1200 object images from ImageNet (*60*), 1000 material images from Flickr Material Database FMD (*61*), and 1000 object or scene images from COCO (*62*). Within ImageNet, we focused on 150 representative object categories, selecting eight images per category. This selection was informed by our previous decoding study (*22*). These images have been validated for training models that demonstrate generalizability in reconstructing artificial shapes (*24*). To enrich the diversity and volume of our training set, we incorporated all images from FMD (eliminating the need for selection) and undertook random sampling from COCO. We also excluded images with a resolution where the width or height was less than 100 pixels. On the basis of preliminary analyses, the inclusion of images from FMD and COCO was found to enhance the quality of reconstruction. Furthermore, it effectively mitigated a bias in which a dominant object often appears at the center of an image. The images were cropped to a square at the center and resized to 500 × 500 pixels.

#### Illusory line images

We created six images with illusory lines induced by offset gratings (fig. S1A). Two gratings were used to fill the gray background image with a predetermined number of equally spaced black lines and a phase shift of half a cycle between the gratings. Hence, there were three parameters: the orientation of the illusory line (illusory orientation), the orientation of the inducer lines (inducer orientation), and the number of inducer lines. We set the number of inducer lines at 19, while varying the illusory orientations (0°, 45°, 90°, and 135°) and inducer orientations (0° and 90°). This resulted in six illusory line images, as the illusory and inducer orientations cannot be the same in a single image. We reduced the number of inducer lines to nine and three for configurations with vertical or horizontal illusory orientation, respectively, creating four control images that induce weaker illusion perception. These parameters were based on previous behavioral studies on human subjects (*28*) and adapted for our experiments. In addition, we included 10 corresponding positive control images with a real line drawn at the location where the illusory line was perceived. Before the fMRI experiments, we displayed stimulus images on the screen in the MRI scanner to the subjects and asked them to report their perceptual experiences without informing them which images were designed to be illusory or control. We verified that the illusory images consistently induced a clear illusion, while the control images induced a weaker or no illusion for all subjects. This was consistently applied to all illusion types, including both the lines and the neon color spreading discussed in the subsequent section.

#### Neon color spreading images

There are two types of neon color spreading images: the Ehrenstein configuration and the Varin configuration. For Ehrenstein (fig. S1B), we prepared four different versions with two sets of lines (four and two) and two sizes (small and large) of the colored portion. The luminance of the colored lines was set between the luminance values of the surrounding black lines and the background (gray) to meet the key requirement for transparency perception (*63*, *64*). The colored lines and the surrounding black lines were connected in the same width, inducing color filling-in perception (transparent color disk). We constructed control images by reducing the width of the black lines, which created disconnected patterns and disrupted the color filling-in perception (*32*). In addition, we added positive control images with uniform color in the expected filling-in areas, while keeping the same black lines as in the illusion images.

For Varin (fig. S1C), the illusion image was composed of four disks, the centers of which could be connected to form a rectangle. Each disk was pieced by a colored 90° sector and a black Pacman. Similar to Ehrenstein, the luminance of the color was made between the luminance values of Pacman (black) and the background (gray). For control, we removed the black Pacman and retained only colored sectors to reduce the color filling-in effect (the main control image used in the analysis of Fig. 4; left in the “control” panel of fig. S1C), which shared the colored lines with the illusion image. Two other control images were created using only black Pacmans or disks. We also prepared two positive control images with uniform color in the rectangular region of interest. The one with the black packmen as in the illusion image was used for the analysis of Fig. 4.

In total, we produced 12 images for Ehrenstein and 6 images for Varin. We selected red for the colored components due to its superior reconstruction quality compared to other colors (*24*). The saturation of the red color in each pattern was adjusted to ensure that all subjects clearly perceived color filling-in (0.8 for four-line and 0.7 for two-line frames of Ehrenstein, and 0.3 for Varin, respectively).

### fMRI experiments

We performed image presentation experiments for two types of sessions: training and testing. All stimuli were rear projected onto a screen in an MRI scanner bore with a luminance-calibrated liquid crystal display projector. The stimulus images were displayed at the screen center with a size of 12° × 12° of visual angle on a gray background. We asked subjects to fixate on the center of the images cued by a circle of 0.3° × 0.3° of visual angle. Each subject used a custom-molded bite bar and/or personalized headcase from CaseForge Inc. to reduce head motion during fMRI data collection. Multiple scanning sessions were performed to collect data for each subject. The total time span of data collection varied among the subjects: approximately 2 years for subjects S1 and S2, 4 years for subjects S3 and S4, and less than 1 year for subjects S5 to S7. Each consecutive session took a maximum of 2 hours, with each run taking 6 to 8 min. The subjects were free to rest adequately between runs or to terminate the experiment at any time.

#### Training session

A single set of training sessions consisted of presenting each of the 3200 natural images once, resulting in 64 runs. Images from different training image sets (ImageNet, FMD, and COCO) were presented in separate runs. There were 32- and 6-s rest periods at the beginning and end of each run, respectively. Each run contained 55 trials, with 50 trials of different images and five randomly interspersed repetition trials that showed the same image as the previous trial. Each image was flashed at 1 Hz during an 8-s trial. We reused the training data of four subjects for ImageNet dataset from previous studies (*24*, *26*), where a rate of 1 Hz was used. We decided to continue to use the same rate for other subjects, as well as for FMD and COCO datasets. There was no rest period between trials. To indicate the onset of a trial, the color of the fixation spot was changed to red 0.5 s before the trial and then switched back to white when the trial began. Subjects were instructed to perform a one-back repetition detection task, in which they were required to press the button if the current stimulus matched the one presented immediately before it. We asked the subjects to maintain steady fixation throughout the run and evaluated their alertness level using one-back task performance. We repeated one set of training sessions five times for subjects S1 to S4 (3200 × 5 = 16,000 training samples) and two times for subjects S5 to S7 (3200 × 2 = 6400 training samples). Before analyzing the illusion test data, we confirmed that training data with fewer repetitions could produce a comparable model performance on an independent test dataset with natural image presentation (*24*). The order of image presentation was randomly assigned across runs.

#### Test session

In the test session, we presented each of the 38 test images (10 illusory line images, 10 real line images, 12 Ehrenstein images, and 6 Varin images) 20 times. For subject S4, we only collected data for 32 test images because of the subject’s relocation before we decided to add the Varin configurations (six images). We included the same number of randomly selected natural images to make the fMRI signal baselines comparable to the training sessions. The test session consisted of 40 runs. Similar to the training session, there were 32- and 6-s rest periods at the beginning and end of each run, respectively. Each run contained 42 trials with 38 trials of different images (19 test images and 19 natural images) and four randomly interspersed repetition trials that showed the same image as the previous trial (subject S4 underwent fewer trials due to the fewer illusion images). To eliminate the aftereffect from the previous test image, we inserted a natural image trial between every two illusion image trials. Otherwise, the presentation order was random in each run. Each image was flashed at 0.625 Hz during an 8-s trial. The slower rate was used in the test session because subjects of preliminary experiments reported that it improved the stability of the illusory perception. For the neon color illusion stimuli (Ehrenstein and Varin), only the colored portions were flashed on black lines or disks to enhance illusory percepts. There was no rest period between trials. The subjects performed a one-back repetition detection task based on the same cue from the fixation spot as in the training session.

### MRI acquisition

We collected fMRI data using a 3.0-T Siemens MAGNETOM Verio scanner at the Kyoto University Institute for the Future of Human Society (formerly, Kokoro Research Center). An interleaved T2*-weighted gradient-echo echo-planar imaging scan was performed to acquire functional images covering the entire brain [repetition time (TR), 2000 ms; echo time (TE), 43 ms; flip angle, 80°; field of view (FOV), 192 × 192 mm^2^; voxel size, 2 × 2 × 2 mm^3^; slice gap, 0 mm; number of slices, 76; and multiband factor, 4]. T1-weighted (T1w) magnetization-prepared rapid acquisition gradient-echo fine-structural images of the entire head were also acquired [TR, 2250 ms; TE, 3.06 ms; inversion time (TI), 900 ms; flip angle, 9°; FOV, 256 × 256 mm^2^; and voxel size, 1.0 × 1.0 × 1.0 mm^3^].

### fMRI data preprocessing

We performed the MRI data preprocessing through the pipeline of FMRIPREP (version 1.2.1). For the functional data of each run, we first estimated a BOLD reference image using a custom methodology of FMRIPREP. Then, data were motion-corrected using MCFLIRT from FSL (version 5.0.9) and slice time-corrected using 3dTshift from AFNI (version 16.2.07), based on this BOLD reference image. Next, we coregistered the corresponding T1w image using boundary-based registration implemented by bbregister from FreeSurfer (version 6.0.1). The coregistered BOLD time-series were then resampled onto their original space (2 × 2 × 2 mm^3^ voxels) with antsApplyTransforms from ANTs (version 2.1.0) using Lanczos interpolation. After obtaining the resampled BOLD time series, we first shifted the time series by 4 s (two volumes) to compensate for hemodynamic delays, and then regressed out nuisance parameters from each voxel’s time series of each run, including a constant baseline, a linear trend, and temporal components proportional to the six motion parameters calculated during the motion correction procedure (three rotations and three translations). We created single-trial data samples by reducing extreme values (beyond ±3 SD for each run) of the time series and averaging within each 8-s trial (four volumes).

### Data exclusion criteria

We fixed the data exclusion criteria before fMRI data collection and finished exclusion before proceeding to the main analyses. First, runs with low performance (hit rate ≤ 50%) in the one-back repetition detection task were excluded. This step was to discard the scanned data when the subject’s alertness level was low. As a result, one run and two runs were discarded from subjects S1 and S2, respectively. Second, runs with large head motion (maximum translation ≥2 mm) were excluded. In this procedure, we excluded two runs from subject S5. After preprocessing the MRI data, we obtained 18 to 20 single-trial samples for each image and subject.

### Brain regions of interest

According to standard retinotopy experiments (*65*, *66*), we delineated V1, V2, V3, and V4. The LOC, FFA, and PPA were identified using conventional functional localizers (*67*—*69*). We defined the higher visual cortex (HVC) region by manually delineating a contiguous region that covered the LOC, FFA, and PPA on the flattened cortical surfaces. The VC was defined by combining V1 to V4 and the HVC.

### DNN image features

We defined the unit activations of a DNN with visual image inputs as stimulus features. For the DNN, we used a variant of AlexNet, BAIR/BVLC CaffeNet model (*34*) pretrained with images in ImageNet to classify 1000 object categories (the pretrained model is available from https://github.com/BVLC/caffe/tree/master/models/bvlc_reference_caffenet). The CaffeNet model has five convolutional layers and three fully connected layers. We resized all stimuli to 227 × 227 pixels before feeding them into the CaffeNet model. We reshaped the outputs of each of the first seven layers (conv1 to conv5, fc6, and fc7 layers; after the rectification operation, if not otherwise stated) to a vector for each visual image. The number of units in each of the CaffeNet layers is as follows: conv1, 209,400; conv2, 186,624; conv3 and conv4, 64,896; conv5, 43,264; and fc6 and fc7, 4096.

### DNN feature decoding

We constructed multivoxel decoders by training a set of linear regression models that predicted stimulus features from multiple fMRI voxel signals induced by the corresponding stimuli, as in previous studies (*22*, *24*, *26*). Using fMRI samples from the training session (training dataset; 16,000 trials for subjects S1 to S4 and 6400 trials for subjects S5 to S7), we trained a distinct decoder for each combination of DNN units and brain areas (whole VC or individual visual subareas). For a target DNN unit, we selected voxels that were most highly correlated (measured using absolute Pearson correlation coefficient) from each brain area based on training data and then provided them to a decoder as inputs (with a maximum of 500 voxels). While the decoder for a specific DNN unit was not trained using the entirety of the brain region, decoders across different DNN units choose varying voxel subsets, potentially covering a large portion of the whole brain region. The weights of a decoder were optimized via least-square minimization with L2 regularization. We set the regularization parameter to 100.

We applied the trained decoders to fMRI data from the test dataset to predict the feature values of individual DNN units (“decoded features”). The decoded features corresponding to a visual image come from a single-trial fMRI sample. We normalized the decoded features for subsequent image reconstruction analyses to compensate for the possible differences in the distributions of the stimulus and decoded features. The variance across normalized decoded features within a layer was matched to the mean variance of DNN feature values, which was calculated from an independent set of 10,000 natural images. The mean of the normalized decoded features was maintained at the same level as that of the unnormalized decoded features.

### Image generator

#### GAN (main method)

To visualize the illusory percepts as images, we adopted a generator network (*35*) that was pretrained using a GAN framework (available from https://lmb.informatik.uni-freiburg.de/resources/binaries). This image generator was trained to transform the rectified outputs of fc6 layer of the CaffeNet model into the original input image using images from ImageNet. We fed the decoded fc6 features from a single-trial or trial-averaged fMRI sample into this generator network to obtain a reconstructed image (*36*). The choice of fc6 aligns with our aim of studying representations of illusory percepts in multiple individual brain areas. The fc6 layer demonstrated decent and comparable linear decodability for all visual areas (*22*). A recent study also found that all ventral stream visual areas corresponded best with layers of similar levels (*70*). We additionally confirmed that fc6 provided more accurate results when mapped to the original image compared to higher layers (*35*). We chose this method for quantitative analyses in this study because the results of pilot research indicated that it tended to produce high-contrast reconstructions, especially for geometric shapes and patterns, compared to other candidate methods.

#### Diffusion

Similar to the main method, we fed the decoded fc6 features to a diffusion-based image generator. Here, we trained a conditional diffusion model after a modification of the model architecture from a previous study (*38*). The diffusion model was trained to generate the original input image conditioned on the rectified outputs of fc6 layer of the CaffeNet model as follows. We used the architecture for the class-conditional ImageNet 64 × 64 model (available from https://github.com/openai/improved-diffusion/), which had a conditioning vector of length 768. To enable training a diffusion model conditioned on the 4096-dimensional fc6 feature vector derived from CaffeNet (see “DNN image features”), we further added a fully connected layer whose input and output sizes were 4096 and 768, respectively. The fc6 features were fed into the diffusion model through this fully connected layer. Approximately 1.2 million natural images from ImageNet (*60*) were used as training images and resized to 64 × 64 pixels. We used the linear noise schedule and set the number of diffusion steps to 4000. We trained the model for 1 million steps using a batch size of 128 and a learning rate of 0.0001.

#### Pixel optimization (iCNN)

In our original deep image reconstruction study (*24*), this method was used to convert decoded features of multiple DNN layers to an image (code available from https://github.com/KamitaniLab/DeepImageReconstruction). The pixel values of an input image were optimized such that its image features matched the decoded features. In the current study implementation, we used CaffeNet as the target of feature decoding for comparison with the other methods. Following (*24*), we used the feature values before the rectification operation from eight layers (conv1 to conv5 and all fully connected layers). We also applied the same loss function and natural image prior and solved the optimization problem using stochastic gradient descent with momentum for 200 iterations.

### Analysis of robustness to noise

To exclude the possibility that stimulus-independent noise in brain-decoded features leads to the reconstruction of illusory components, we added noise to stimulus fc6 features and fed them into the generator. Lacking prior knowledge of the noise distribution, we adopted a nonparametric method to sample the noise. We assumed that the noise for an individual DNN unit follows the same unknown probability distribution across the nonillusory trials of the same subject (individual units do not necessarily share the same distribution). The following analysis was performed separately for each subject. We calculated the empirical noise distributions by pooling the differences between decoded and stimulus features across nonillusory trials. We then randomly sampled the noise value from the empirical distribution for each DNN unit and added them to the stimulus features of an illusory image.

### Evaluation of reconstruction

#### Illusory line

We evaluated each single-trial reconstructed image of the line illusion, both globally (whole image) and locally (restricted to a specific image region). For the local case, we investigated two types of image regions: illusory and nonillusory. To delineate the regions for each stimulus image, we cropped the four largest disks tangent to the middle lines of the image, among which two disks consisted of illusory lines and two disks did not (Fig. 3E). We detected the principal orientation for each image/region of interest and compared which of the illusory and inducer orientations was more similar to the principal orientation, using cosine similarity. The principal orientation was detected using the method that has been used in texture analysis (*44*). We converted the images into grayscale and applied Radon transform to detect the linear trends. More specifically, the largest disk area *A* in the image region was selected and projected to a line space by summing the pixel intensities along each line within *A*

$$R(r,\theta )=\sum_{(x,y)\in A}I(x,y)\delta (r-x\cos\theta -y\sin\theta )$$

where each line is parametrized by the distance from the center *r* and the orientation θ. The intensity of the pixel located at (*x*, *y*) is denoted by *I*(*x*, *y*). If (*x*, *y*) is on the line, δ = 1, otherwise, δ = 0.

For each orientation, we calculated the variance of the projections across lines that intersect a small disk region in the center of the image. Intuitively, a prominent black line at a specific orientation would result in a substantial decrease in the projection value, causing a sharp change in projections across the neighboring lines of the same orientation. We calculated this variance for different orientations and defined the principal orientation as the one with the largest variance. Only the lines close (no more than five pixels in distance) to the center of the region were used to calculate variance. This restricted range was expected to contain the illusory line.

#### Illusory color

To evaluate the color filling-in effect, we performed a regression analysis in which a reconstructed image was approximated by the superposition of the illusory surface and the inducing stimulus with an additive error term. Given the real color surface in the positive control image, we performed another regression analysis that excluded the inducing stimulus regressor to compare the illusory color with the real color.

Before the regression analysis, we created redness maps from the reconstructions, the illusory/real surfaces, and the inducing stimulus. A redness map was generated by converting the RGB image to the HSV color space, using version 4.5.2 of the openCV library, and by extracting the saturation (*S*) values of the red pixels. The nonred pixels were assigned a value of zero. The red pixels were identified as those with hue values (*H*) in the range of 0° to 10°, or 160° to 180°. The regression models for the redness map of each single-trial reconstruction were as follows

$$y_{i}=\varepsilon_{i}+\beta_{1}x_{i,1}+\beta_{2}x_{i,2}\;(\text{illusion versus control})$$

$$y_{i}=\varepsilon_{i}+\beta_{1}x_{i,1}\;(\text{illusion versus positive control})$$

with *y_i_* (*i* = 1,2, …, *h* × *w*) the value of the *i*-th pixel in the redness map of a reconstruction of size *h* × *w*, ɛ*_i_* the error term, *x*_*i*,1_ and *x*_*i*,2_ the values of the *i*-th pixel in the redness maps of the illusory/real surface and the inducing stimulus, β_1_ and β_2_ the coefficients for the illusory/real surface and the inducing stimulus. The coefficients were estimated by minimizing the sum of squared errors across the pixels in each reconstruction.

### Analysis of individual units’ responses

#### Orientation selectivity

To examine whether individual DNN units could respond similarly to real and illusory line orientations, we introduced illusory images comprised of concentric curves broken at the center, along with their corresponding positive control images with a central real line (fig. S4A). The spacing between adjacent concentric curves was kept constant, and the phases of the concentric halves were randomly assigned from a range of 0 to 1 cycle. To balance the background curves, the images with flipped phases between two parts were paired, and the averaged activation was used to evaluate orientation selectivity.

We first identified units selective to a specific orientation using a real central line of 12 different orientations (in 15° steps) on unbroken concentric patterns with phases matched with the illusion/positive control image. The concentric patterns were presented in the background because unit activation to a central orientation depended on the background context, especially in the middle and higher layers. Among center-responsive units [the center units of each channel in convolutional layers (864 for conv1, 256 for conv2 and conv5, and 384 for conv3 and conv4) and all units in fully connected layers], we selected the units that exhibited higher activation to the orientation of interest than to all the other orientations for both of the background phases. We then ranked them based on the difference in activation between the orientation of interest and the other orientations. Using the top 5% units of each layer, we calculated the activations to all 12 orientations for the illusion and the positive control images. We repeated this procedure for 50 randomly assigned pairs of background phases.

#### Color (hue) selectivity

To examine whether individual DNN units could respond similarly to real and illusory colors, we identified units responsive to real color (fig. S5A) and then compared their activations to the illusion, the control, and the positive control images (fig. S5B; see “visual stimuli”).

We first identified units selective to red color, using a uniform surface (disk or square) with three levels of luminance (0.3, 0.5, and 1, relative to the luminance of the image background color based on measurements of the display) and four levels of saturation (0, 0.3, 0.7, and 1) on the inducer patterns. Among the units that showed higher activations to red (nonzero saturation) than to gray (zero saturation) for all the luminance and saturation levels, we ranked the units by the difference in activation averaged across all comparisons between the red and gray surfaces. The activations for the top 5% units are shown for each layer after subtracting the activation to the gray surface.

### Statistical tests and sample size

In this study, each subject was regarded as a replication unit (*71*); thus, statistical tests were primarily conducted on a per subject basis. The sample size of test data (the number of trials for each image/condition) was determined before the test experiments.

In the analysis of principal orientations, we planned to perform one-sided *z* tests to determine whether the closer-to-illusory proportion was greater in one condition than in the other. To achieve a statistical power of 80%, at least 20 samples are required for each condition to detect a large effect size (Cohen’s *h* = 0.8) at a significance level of 0.05. Thus, we repeated 20 trials for each stimulus image in each subject. In the present paper, we only present the results of statistical tests on the trials pooled across different stimulus configurations. Thus, one condition for statistical comparison involves more than 20 trials. The numbers of trials that survived the exclusion criteria for the results in Fig. 3C to compare decoded features (*n*_1_) and stimulus features (*n*_2_) were *n*_1_ = *n*_2_ = 117, 116, 120, 120, 113, 120, and 120 for subjects S1 to S7, respectively, and in Fig. 3 (D and F) to compare local illusory (*n*_1_) and nonillusory (*n*_2_) regions were *n*_1_ = *n*_2_ = 234, 232, 240, 240, 226, 240, and 240 for subjects S1 to S7.

In the analysis of illusory color, we planned to perform one-sided *t* tests on the illusory surface coefficients obtained from individual trials/reconstructions. To achieve a statistical power of 80%, at least 20 samples are required for each condition to detect a large effect size (Cohen’s *d* = 0.8) at a significance level of 0.05. Thus, we repeated 20 trials for each stimulus image in each subject. The actual numbers of trials that survived the exclusion criteria in Fig. 4 (E and F) to compare the illusion (*n*_1_) and the control (*n*_2_) conditions were *n*_1_ = 77, 74, 80, 80, 75, 80, 80 and *n*_2_ = 79, 75, 80, 80, 77, 80, 80 (subjects S1 to S7) for Ehrenstein, and *n*_1_ = 20, 20, 20,19, 20, 20 and *n*_2_ = 20, 20, 20, 19, 20, 20 (subjects S1 to S3 and S5 to S7) for Varin. The same numbers of trials/reconstructions were used for the results in Fig. 4 (I and J).
