# Supplementary material for: Reconstructing visual illusory experiences from human brain activity
Source: Sci Adv. 2023 Nov 15;9(46):eadj3906. doi: 10.1126/sciadv.adj3906 (PMC10651116; doi:10.1126/sciadv.adj3906)
Supplement: Supplementary file 1 — Figs. S1 to S23 Legends for movies S1 to S3 [file sciadv.adj3906_sm.pdf]

Science Advances
AAAS

Supplementary Materials for

Reconstructing visual illusory experiences from human brain activity

Fan L. Cheng *et al.*

Corresponding author: Fan L. Cheng, chengfanbrain@gmail.com; Yukiyasu Kamitani, kamitani@i.kyoto-u.ac.jp

Sci. Adv. **9**, eadj3906 (2023)
DOI: 10.1126/sciadv.adj3906

The PDF file includes:

Figs. S1 to S23
Legends for movies S1 to S3

Other Supplementary Material for this manuscript includes the following:

Movies S1 to S3

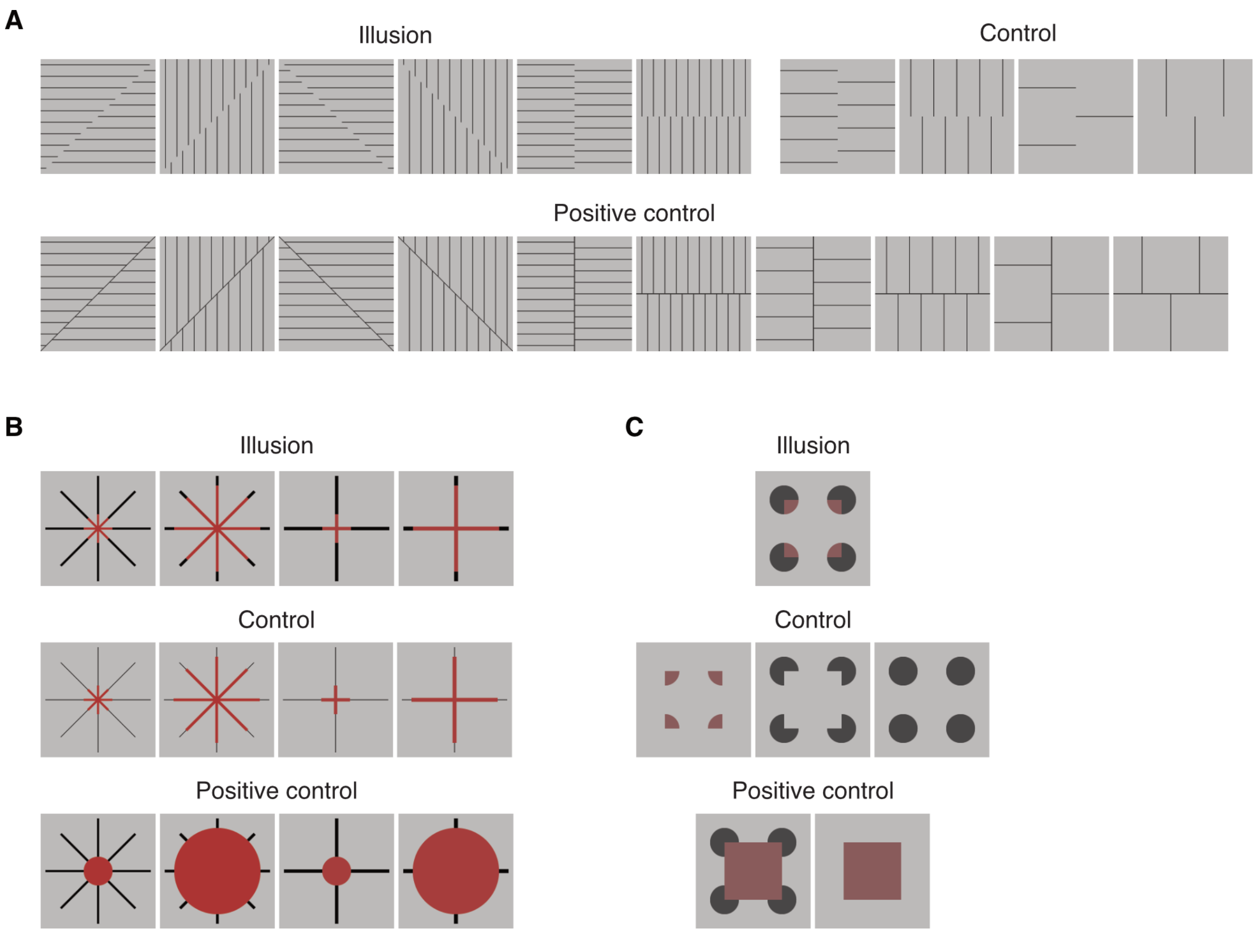

Fig. S1. Test images used in fMRI experiment. (A) Example illusory and positive control images with the central line of different orientations. **(B)** Ehrenstein configuration for neon color spreading. **(C)** Varin configuration for neon color spreading.

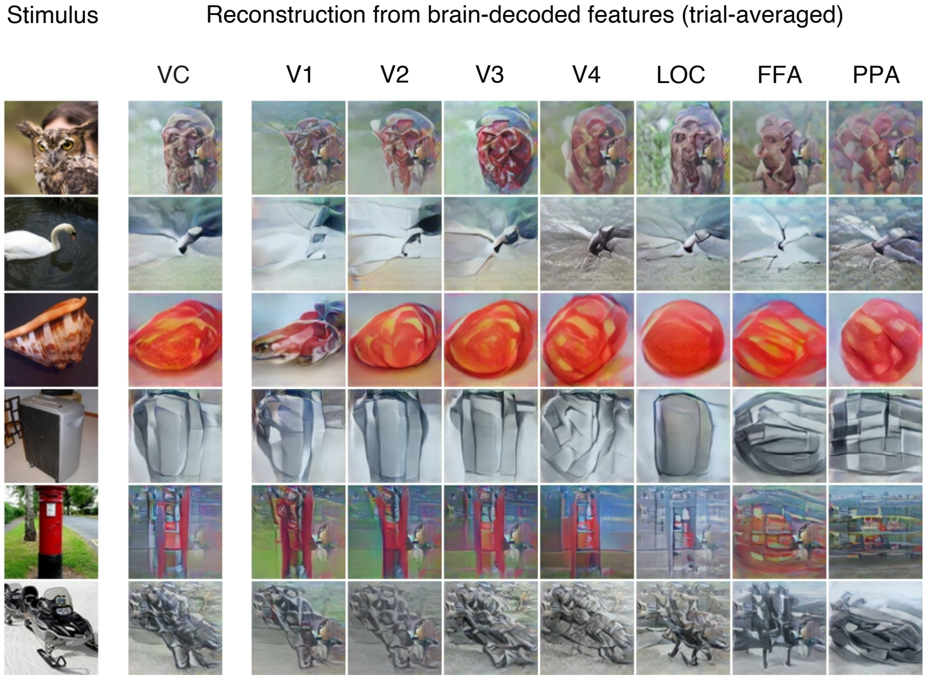

Fig. S2. Reconstructions of natural images from brain-decoded features. The results of each column were produced from averaged fMRI signals across 24 trials in the whole visual cortex (VC) and individual visual areas of subject S2.

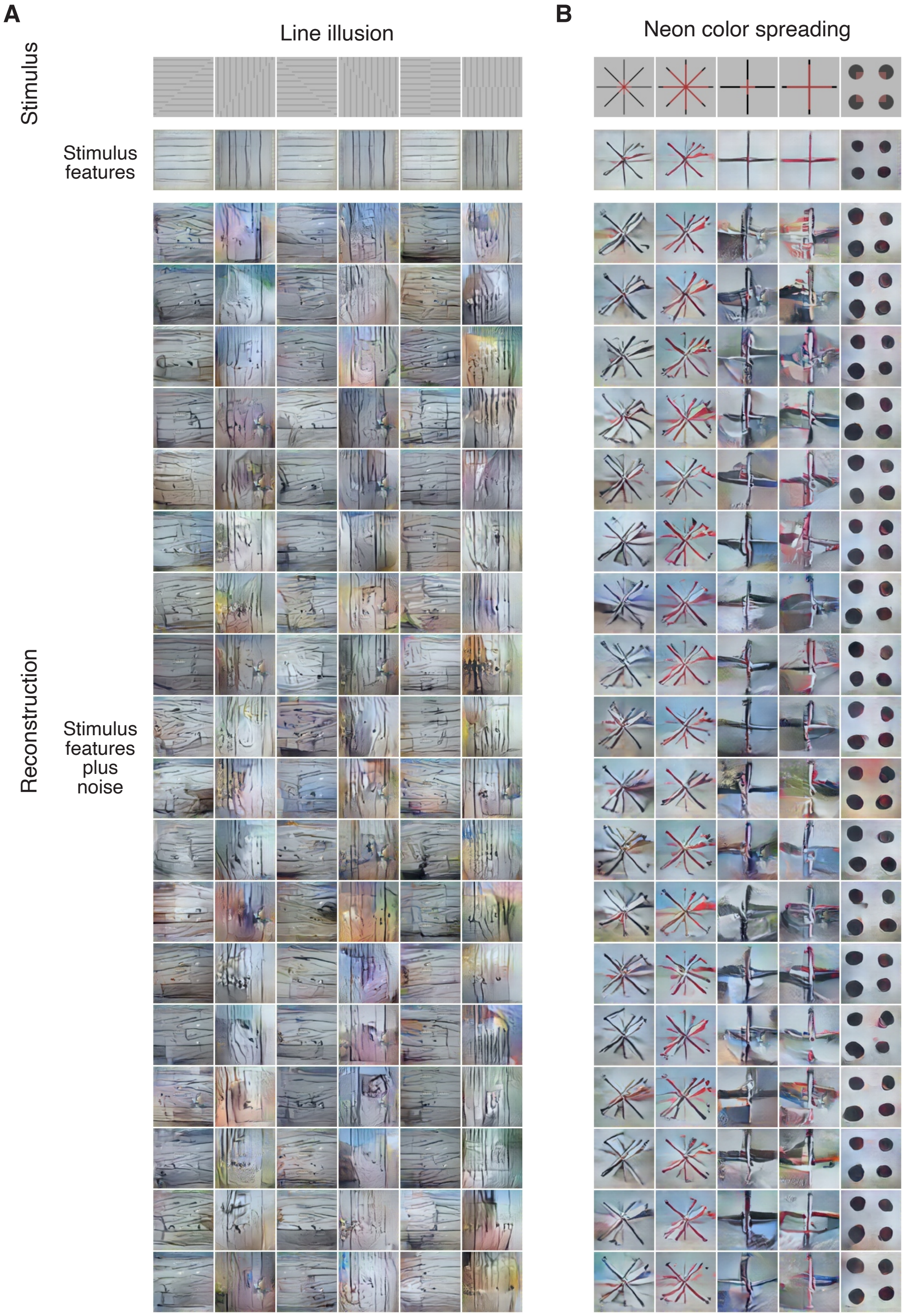

Fig. S3. Reconstructions of illusory images from stimulus features plus the noise. The noises were sampled from the empirical noise distribution computed from the brain-decoded features of non-illusory images. **(A)** Line illusion. **(B)** Neon color spreading.

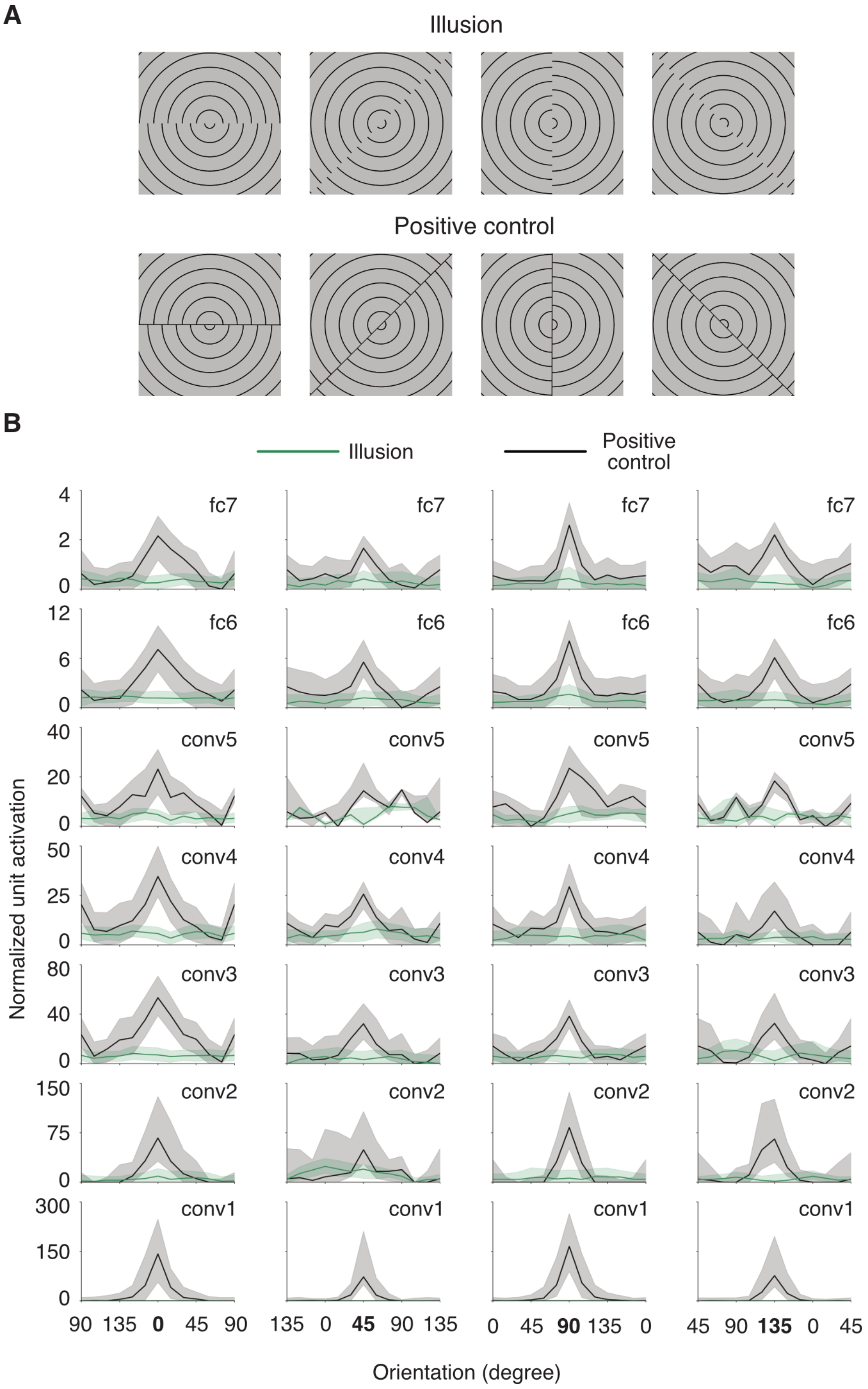

Fig. S4. Individual DNN units do not show orientation tuning shared between illusory and real lines. (A) Illusory line induced by offset-gratings. **(B)** Tuning curves of the orientation-selective units. The tuning curve of each unit was normalized by subtracting the minimum activation value of all orientations. Lines represent the median activation and shaded areas represent the interquartile range of the units pooled across different background phases. The tuning

curves showed sharp peaks in the units' preferred orientation for the positive control images (black) but not for the illusory images (green). The robustness of the results was confirmed when the central line of the positive control was white.

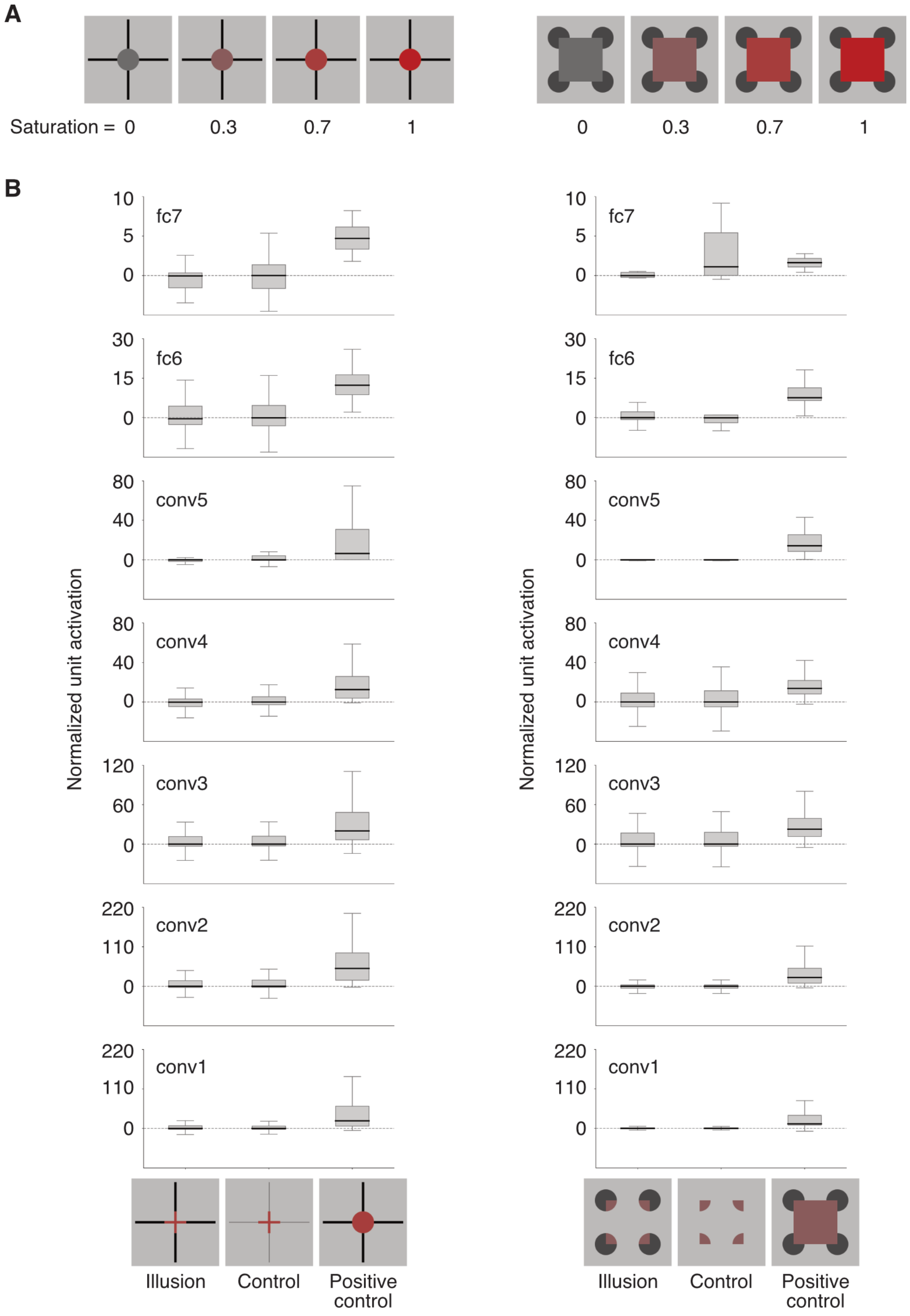

Fig. S5. Individual DNN units do not respond to illusory color similar to real color. Units were analyzed for Ehrenstein (left) and Varin (right), respectively. **(A)** Example images used to identify red-sensitive units. **(B)** Normalized activation of color-selective units. Black lines represent the median value and shaded areas represent the interquartile range of units. If units respond to illusory color, the plots should show similar values for illusion and positive control

images, which are larger than the control condition. However, illusion and control are almost identical, supporting the idea that units do not respond to illusory color. This small difference was not due to the lack of sensitivity of the units. In most units, control and illusion showed identical activation, or control showed relatively higher activation, not the other way around.

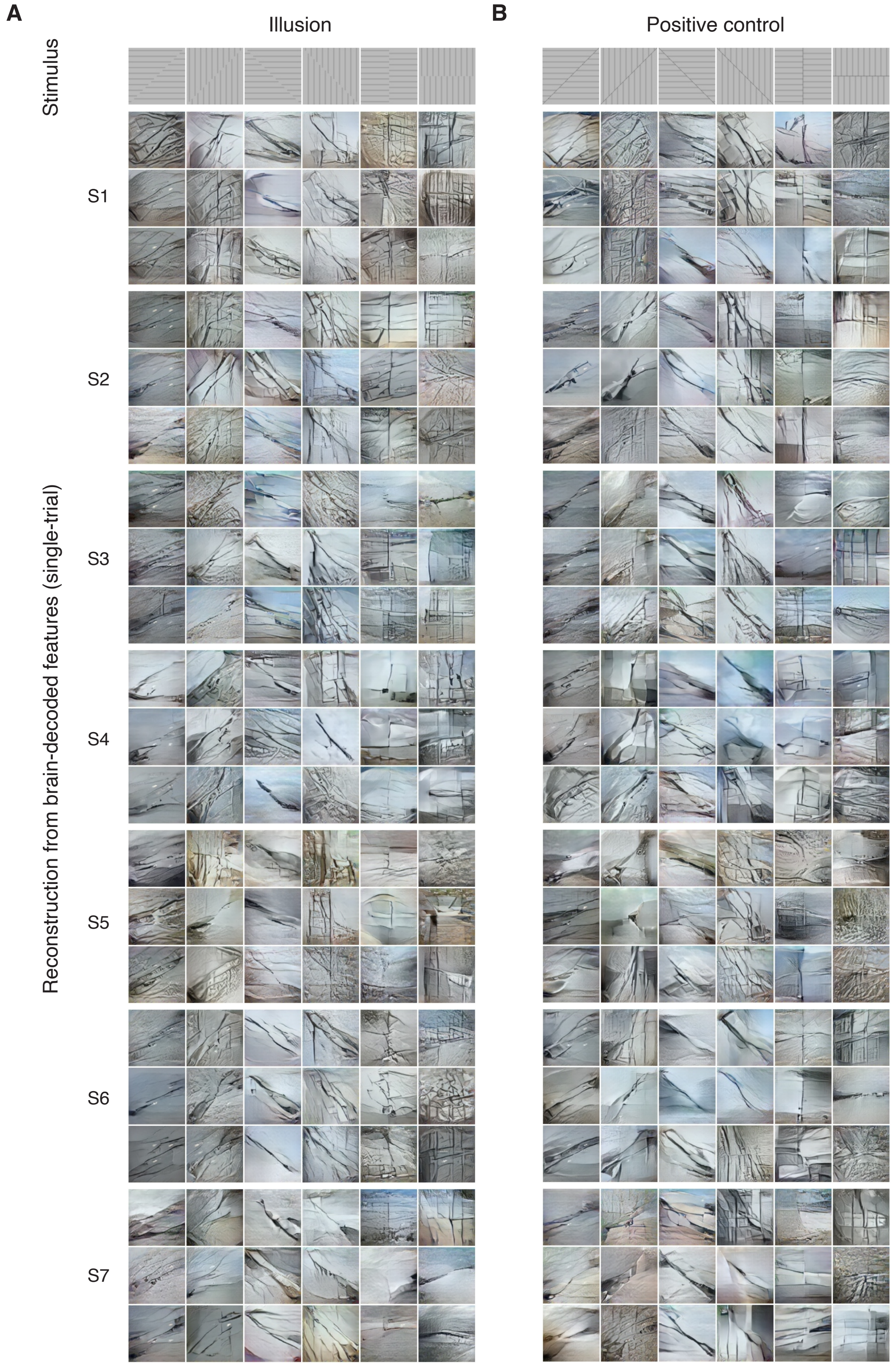

Fig. S6. Reconstructions of line illusion for different configurations from brain-decoded features. Results were produced from single-trial fMRI signals in the whole visual cortex (VC).

Representative reconstructions from three different trials are shown from each subject (no overlapping trials with Figure 2). **(A)** Illusion condition. **(B)** Positive control condition.

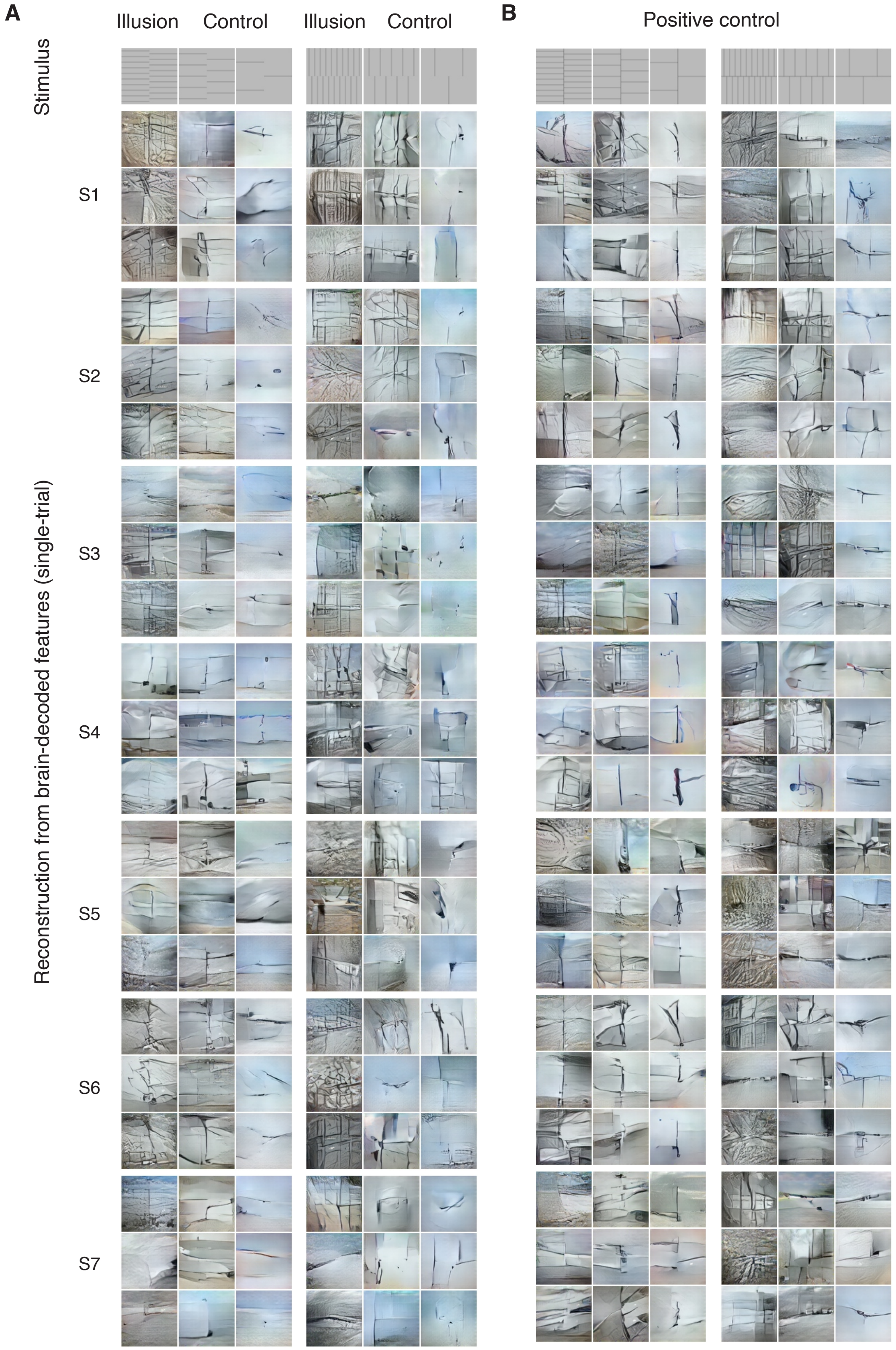

Fig. S7. Reconstructions of illusory and control images from brain-decoded features for line illusion. Results were produced from single-trial fMRI signals in the whole visual cortex (VC).

Representative reconstructions from three different trials are shown from each subject (no overlapping trials with Figure 2). **(A)** Illusion and control condition. **(B)** Positive control condition.

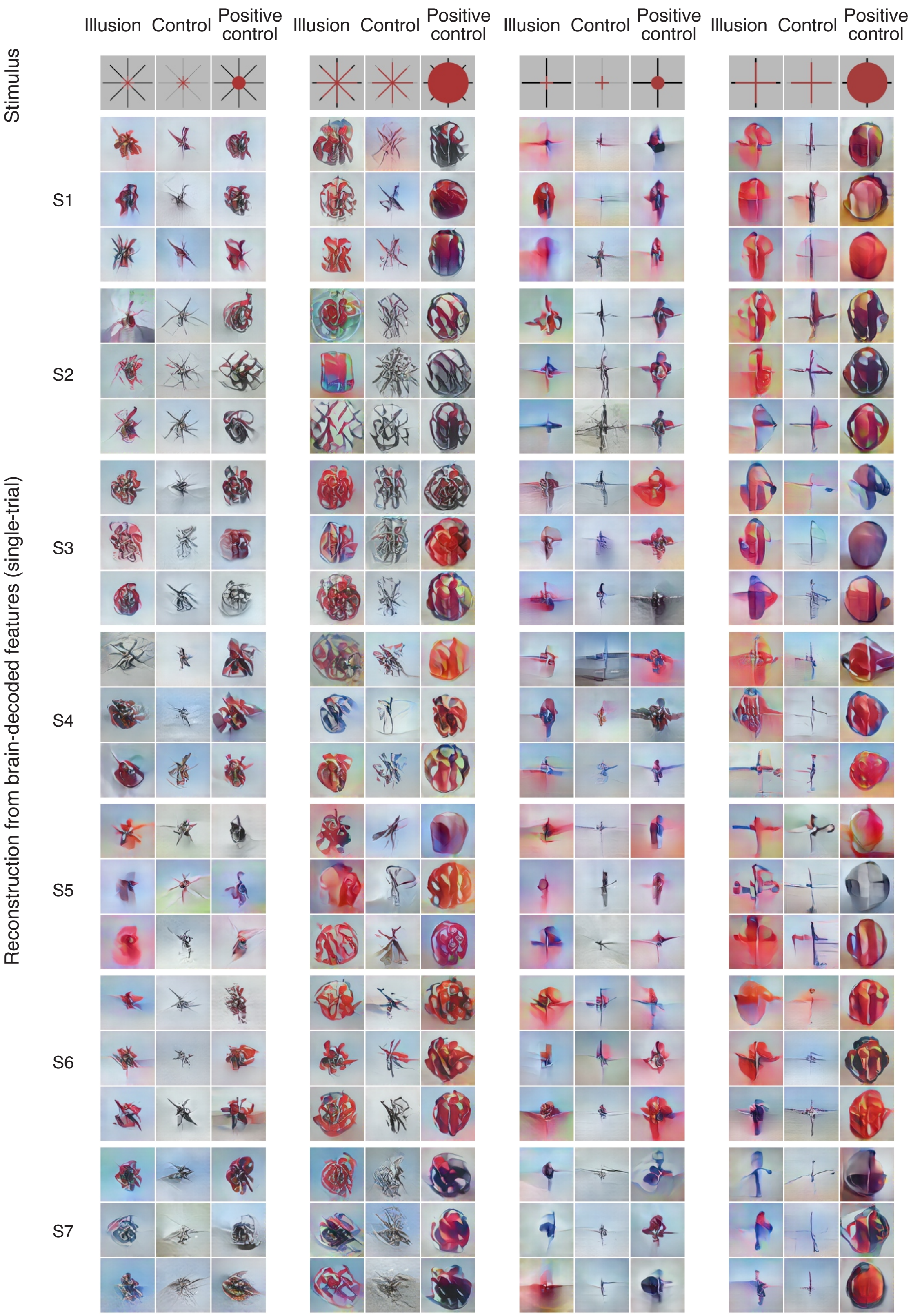

Fig. S8. Reconstructions of neon color spreading (Ehrenstein) from brain-decoded features. Results were produced from single-trial fMRI signals in the whole visual cortex (VC).

Representative reconstructions from three different trials are shown from each subject (no overlapping trials with Figure 2). Every three columns show illusion (left), control (middle), and positive control (right) conditions of the same configuration.

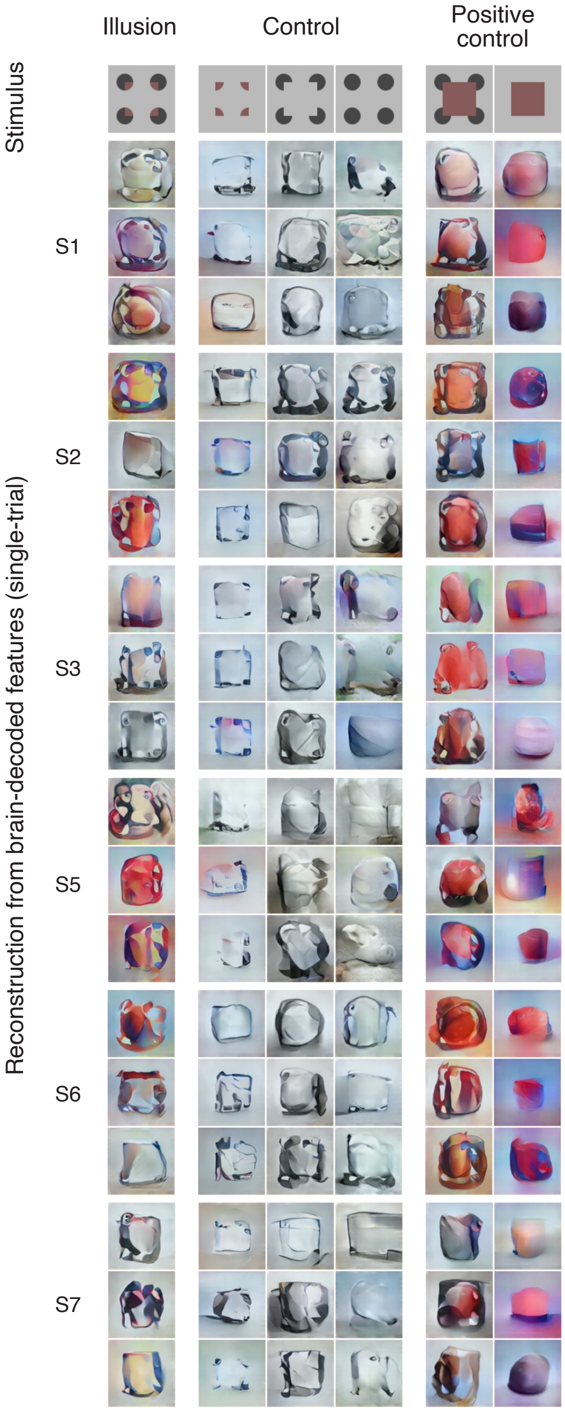

Fig. S9. Reconstructions of neon color spreading (Varin) from brain-decoded features. Results were produced from single-trial fMRI signals in the whole visual cortex (VC). Representative reconstructions from three different trials are shown from each subject (no overlapping trials with Figure 2). The three panels show illusion (left), control (middle), and positive control (right) conditions.

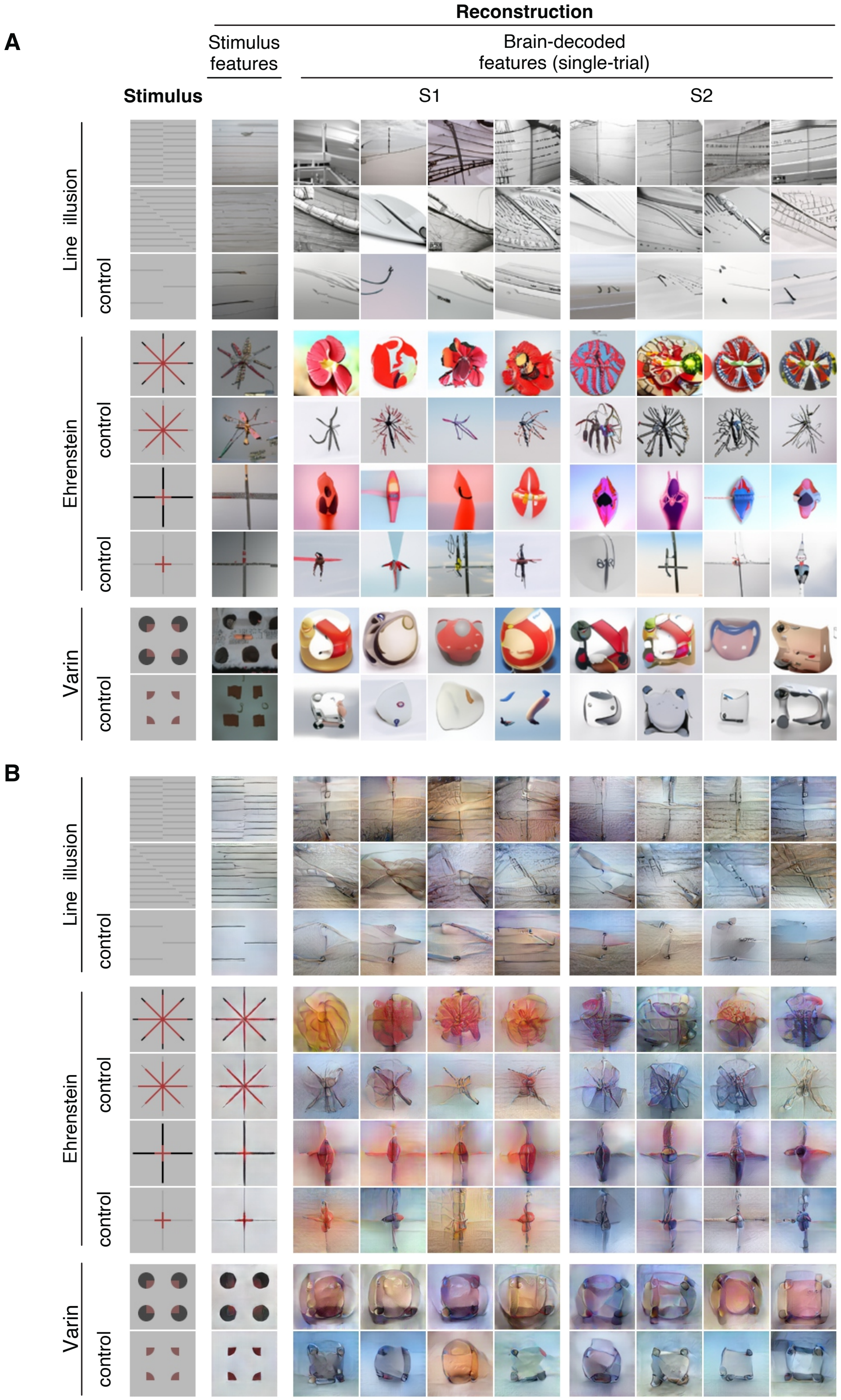

Fig. S10. Reconstructions of illusory and control images using other generators. Reconstructions from stimulus features and from brain-decoded features are shown for two

representative subjects (S1, S2). Reconstructions from brain- decoded features were produced from single-trial fMRI signals (same trials as those shown in Figure 2) in the whole visual cortex (VC). **(A)** Diffusion. **(B)** Pixel optimization (iCNN).

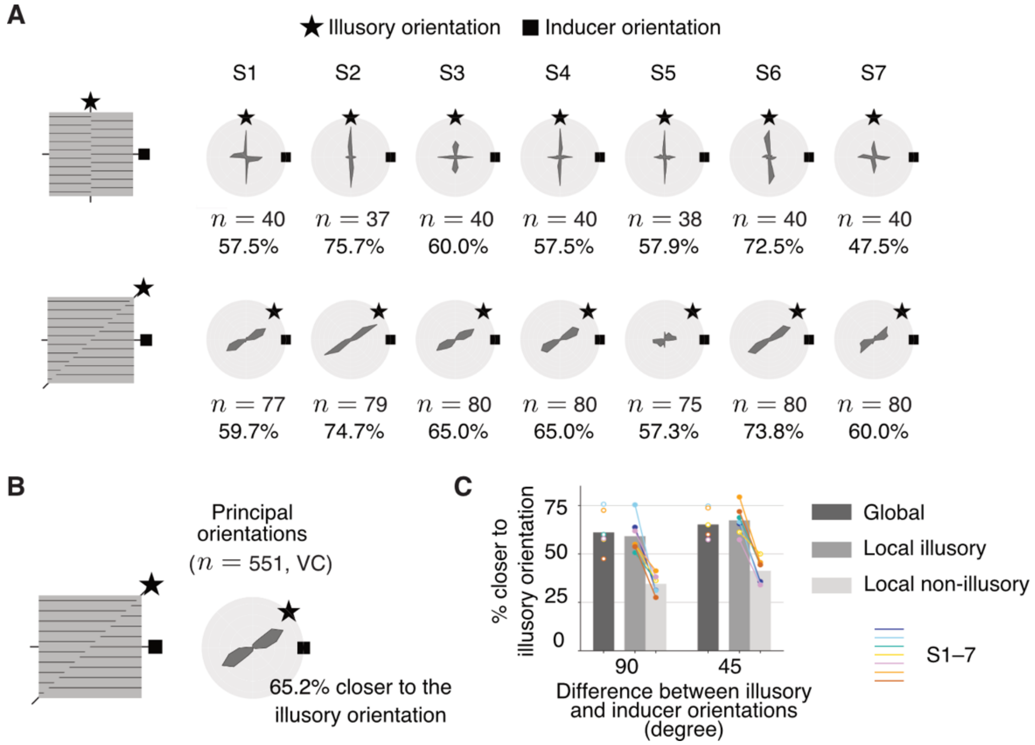

Fig. S11. Evaluation of line illusion reconstructions for 90°- and 45°-difference configurations. Results are based on single-trial reconstructions from VC. (**A**) Distributions of principal orientations in reconstructions from individual subjects. The results pooled across 90°- (top) and 45°- (bottom) difference configurations are shown for each subject (totalling n samples; bin size = 15°). (**B**) Distribution of principal orientations in reconstructions for 45°-difference configurations (pooled across seven subjects, totalling n samples; bin size = 15°). (**C**) Proportions of principal orientations closer to the illusory than the inducer orientation. Color circles and lines indicate individual subjects. Comparisons with a statistically significant difference at the individual level are marked by solid circles.

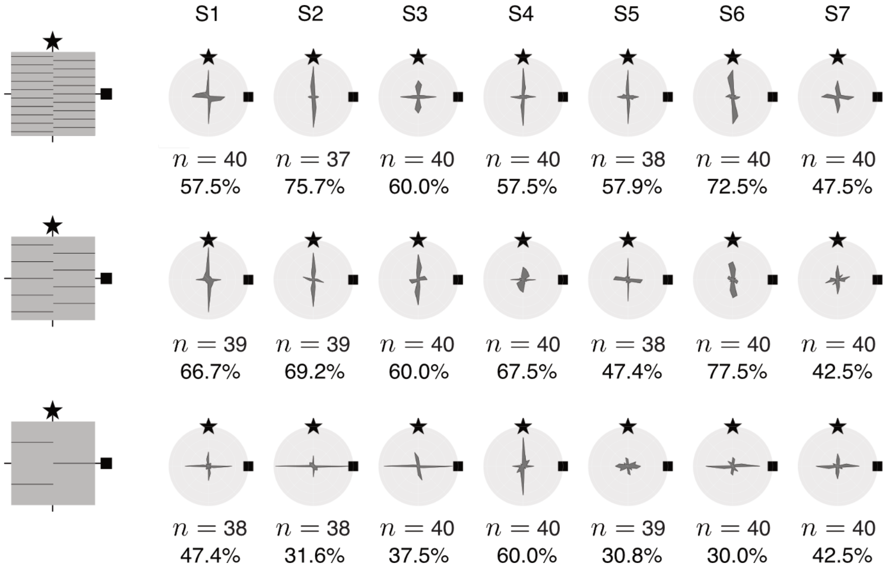

Fig. S12. Evaluation of line illusion reconstructions for different numbers of inducer lines. Distributions of principal orientations in single-trial reconstructions from VC (results for 90°-difference configurations are pooled for each subject, totalling $\boldsymbol{n}$ samples; bin size = 15°).

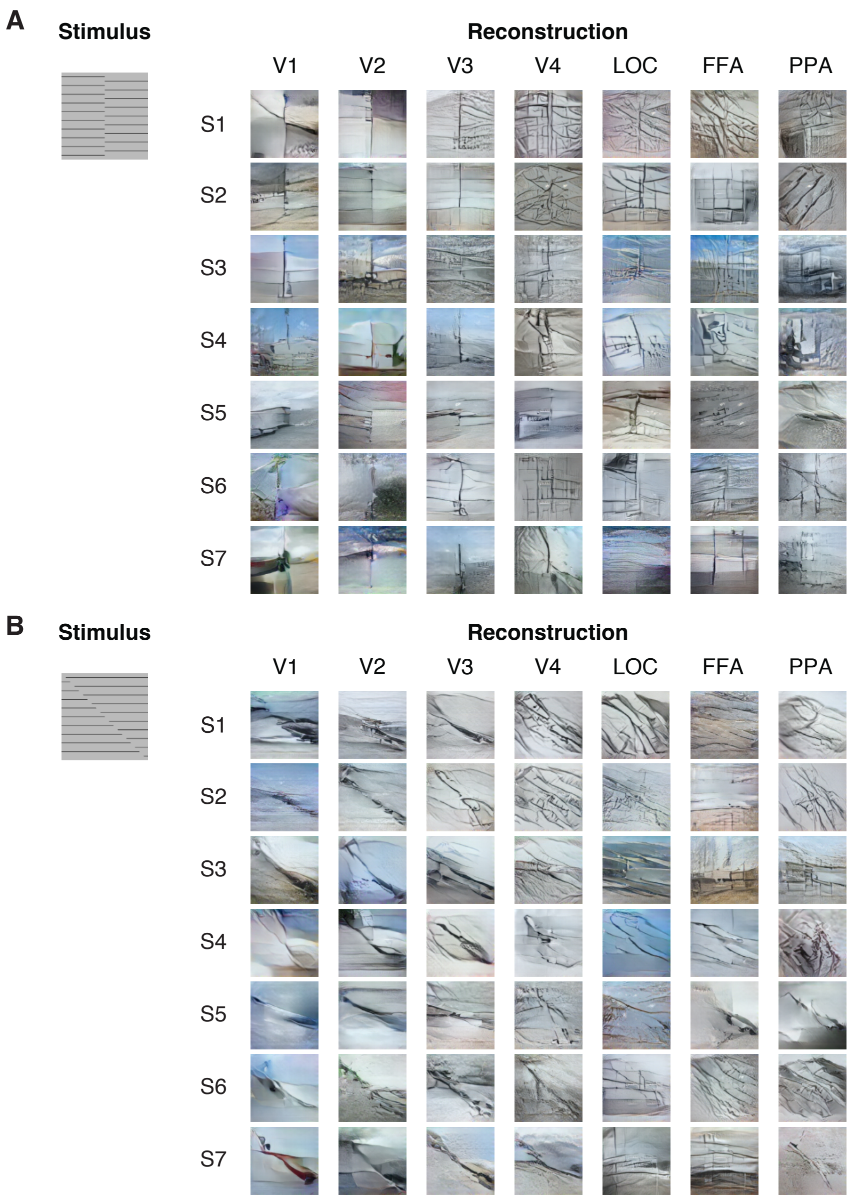

Fig. S13. Reconstructions of line illusion from individual visual areas. Representative reconstructions from single-trial brain activity are shown for each subject (no overlapping trials with Figure 3). **(A)** 90°-difference configuration. **(B)** 45°-difference configuration.

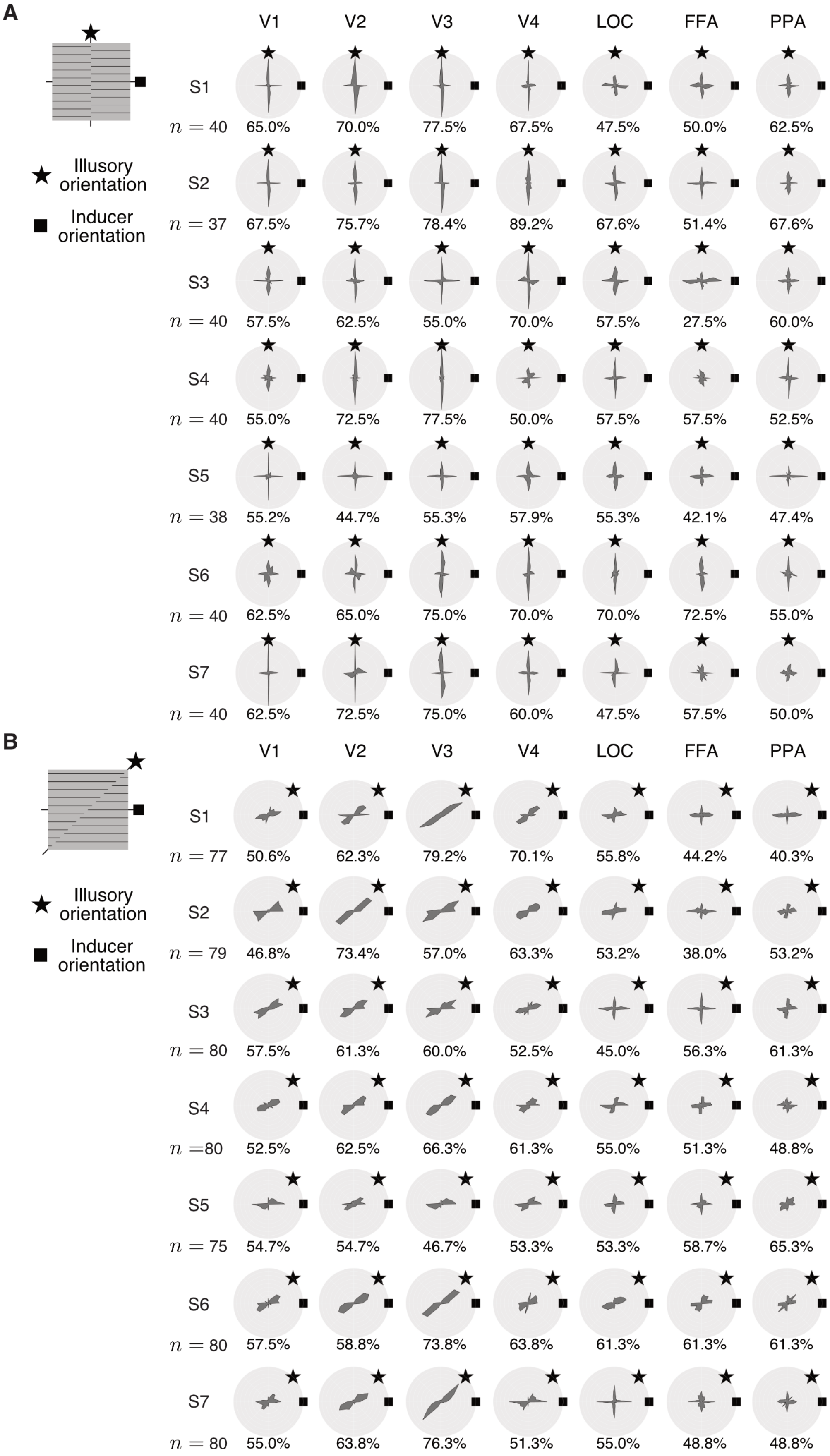

A
V1 V2 V3 V4 LOC FFA PPA
Illusory orientation
Inducer orientation
S1 n = 40 65.0% 70.0% 77.5% 67.5% 47.5% 50.0% 62.5%
S2 n = 37 67.5% 75.7% 78.4% 89.2% 67.6% 51.4% 67.6%
S3 n = 40 57.5% 62.5% 55.0% 70.0% 57.5% 27.5% 60.0%
S4 n = 40 55.0% 72.5% 77.5% 50.0% 57.5% 57.5% 52.5%
S5 n = 38 55.2% 44.7% 55.3% 57.9% 55.3% 42.1% 47.4%
S6 n = 40 62.5% 65.0% 75.0% 70.0% 70.0% 72.5% 55.0%
S7 n = 40 62.5% 72.5% 75.0% 60.0% 47.5% 57.5% 50.0%
B
V1 V2 V3 V4 LOC FFA PPA
Illusory orientation
Inducer orientation
S1 n = 77 50.6% 62.3% 79.2% 70.1% 55.8% 44.2% 40.3%
S2 n = 79 46.8% 73.4% 57.0% 63.3% 53.2% 38.0% 53.2%
S3 n = 80 57.5% 61.3% 60.0% 52.5% 45.0% 56.3% 61.3%
S4 n =80 52.5% 62.5% 66.3% 61.3% 55.0% 51.3% 48.8%
S5 n = 75 54.7% 54.7% 46.7% 53.3% 53.3% 58.7% 65.3%
S6 n = 80 57.5% 58.8% 73.8% 63.8% 61.3% 61.3% 61.3%
S7 n = 80 55.0% 63.8% 76.3% 51.3% 55.0% 48.8% 48.8%

Fig. S14. Evaluation of line illusion reconstructions from individual visual areas. Distributions of principal orientations in single-trial reconstructions are shown for each subject (pooled across all 90°- or 45°-difference configurations, totalling n samples; bin size = 15°). (**A**) 90°-difference configuration. (**B**) 45°-difference configuration.

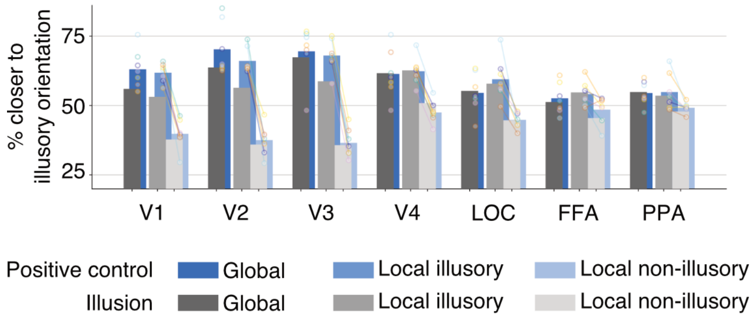

Fig. S15. Comparison between positive control and illusion conditions for line illusion. Proportions of principal orientations closer to the illusory than to the inducer orientation are shown for individual visual areas (pooled across all subjects and configurations; gray bars are the same as that in Figure 3F). Color circles and lines indicate individual subjects for the positive control condition.

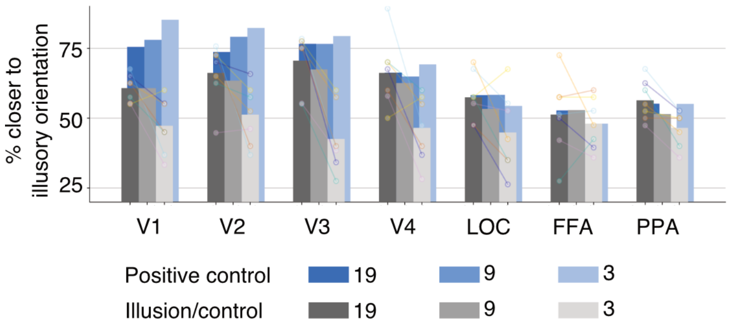

Fig. S16. Comparison between different numbers of inducer lines for line illusion. Proportions of principal orientations closer to the illusory than to the inducer orientation in global regions are shown for individual visual areas. Color circles and lines indicate individual subjects for illusion or control conditions.

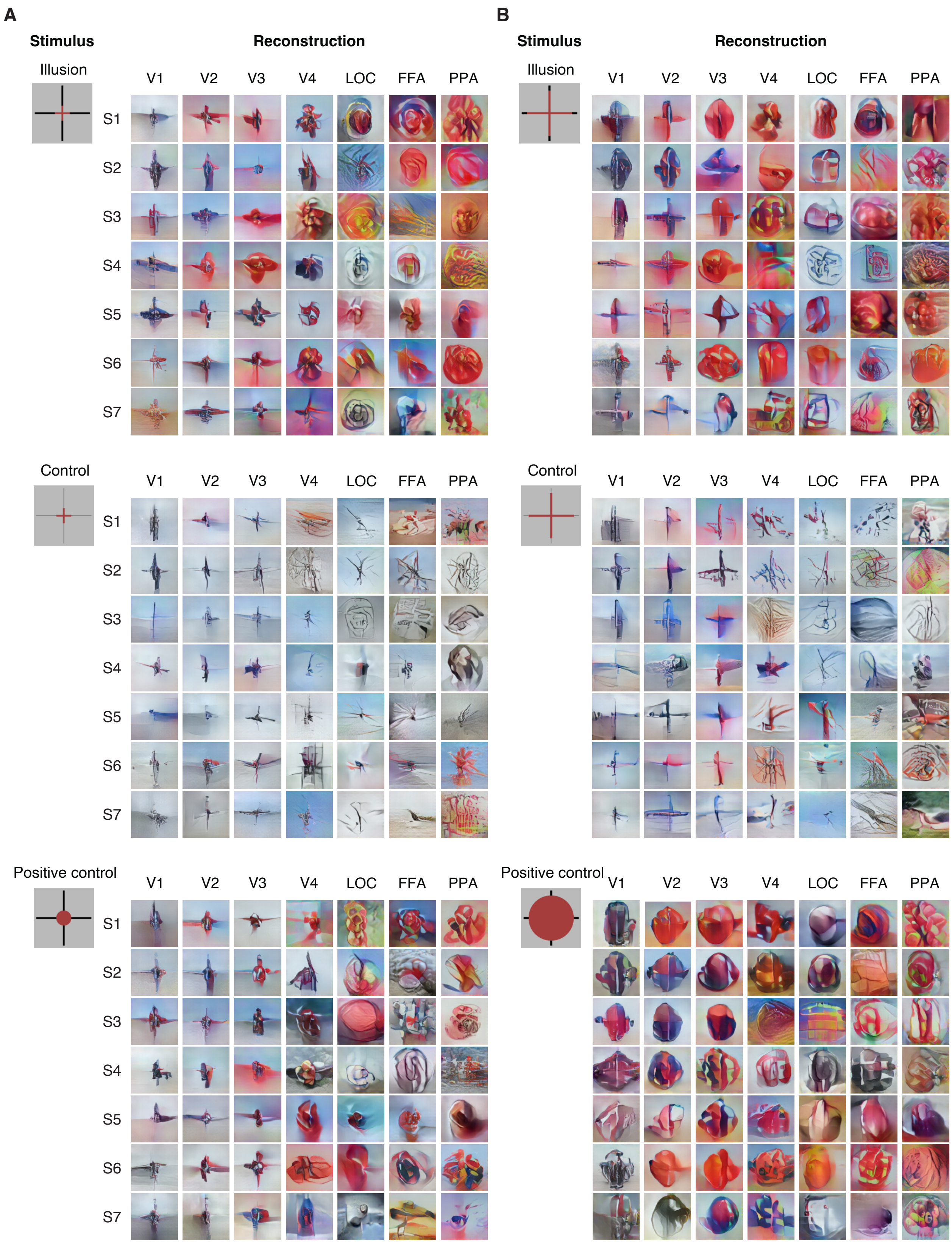
A
Stimulus
Reconstruction
Illusion
V1
V2
V3
V4
LOC
FFA
PPA
S1
S2
S3
S4
S5
S6
S7
Control
Positive control
B

Fig. S17. Reconstructions of neon color spreading (Ehrenstein) from individual visual areas. Representative single-trial reconstructions of the illusion (top), control (middle), and positive control (bottom) conditions are shown for each subject (no overlapping trials with Figure 4). **(A)** Small size. **(B)** Large size.

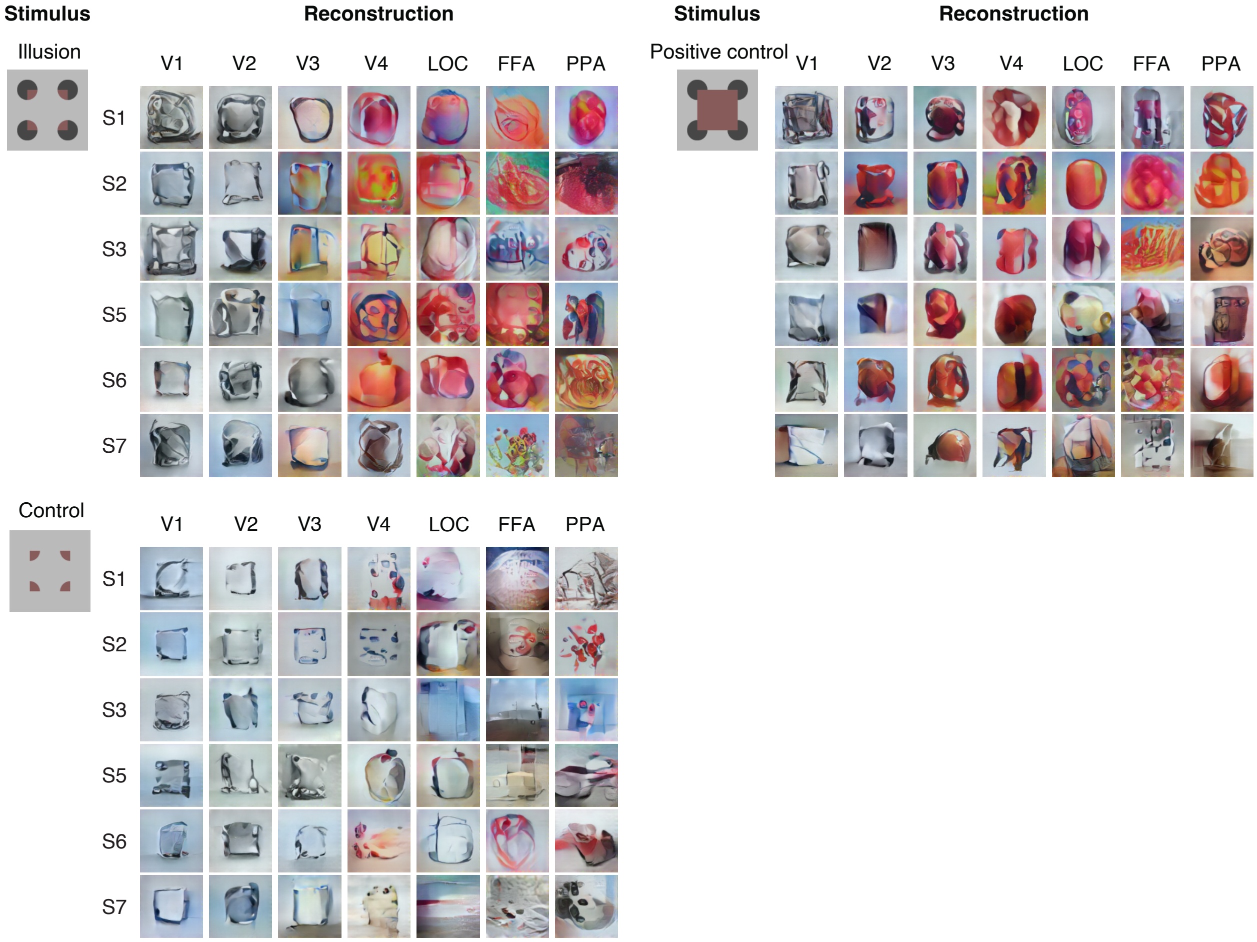

Fig. S18. Reconstructions of neon color spreading (Varin) from individual visual areas. Representative single-trial reconstructions of the illusion (left top), control (left bottom), and positive control (right) conditions are shown for each subject (no overlapping trials with Figure 4).

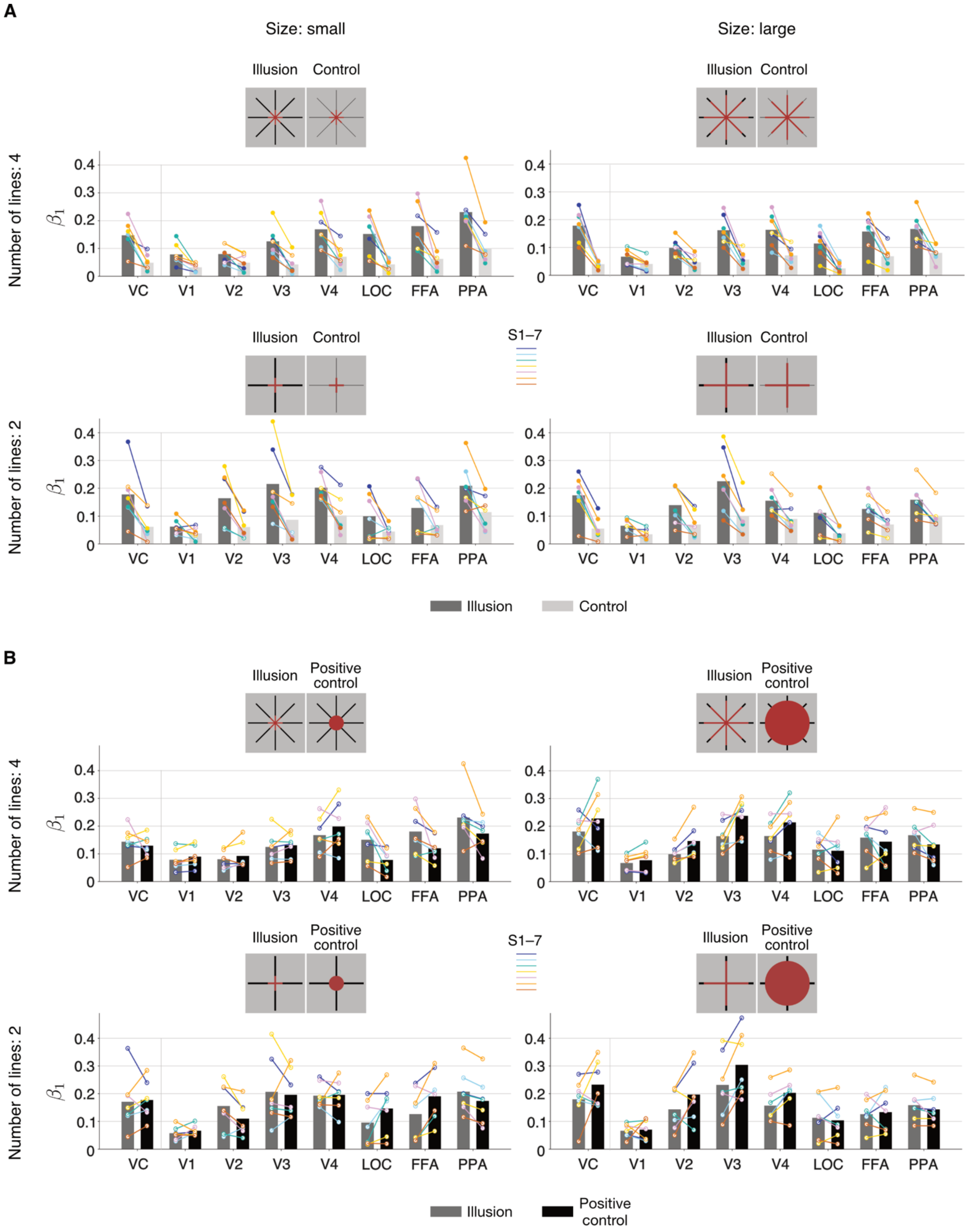
A
Size: small
Size: large
Illusion
Control
Number of lines: 4
Number of lines: 2
β_1
0.4
0.3
0.2
0.1
0
VC
V1
V2
V3
V4
LOC
FFA
PPA
S1–7
Illusion
Control
B
Illusion
Positive control
Number of lines: 4
Number of lines: 2
β_1
VC
V1
V2
V3
V4
LOC
FFA
PPA
S1–7
Illusion
Positive control

Fig. S19. Evaluation of neon color spreading (Ehrenstein) reconstructions for different sizes and numbers of lines. Results are based on single-trial reconstructions from VC and individual visual areas. Color circles and lines indicate individual subjects. **(A)** Comparison of the illusory surface coefficient values between illusion and control conditions. Comparisons with a statistically significant difference at the individual level are marked by solid circles. **(B)** Comparison of the illusory surface coefficient values between illusion and positive control conditions.

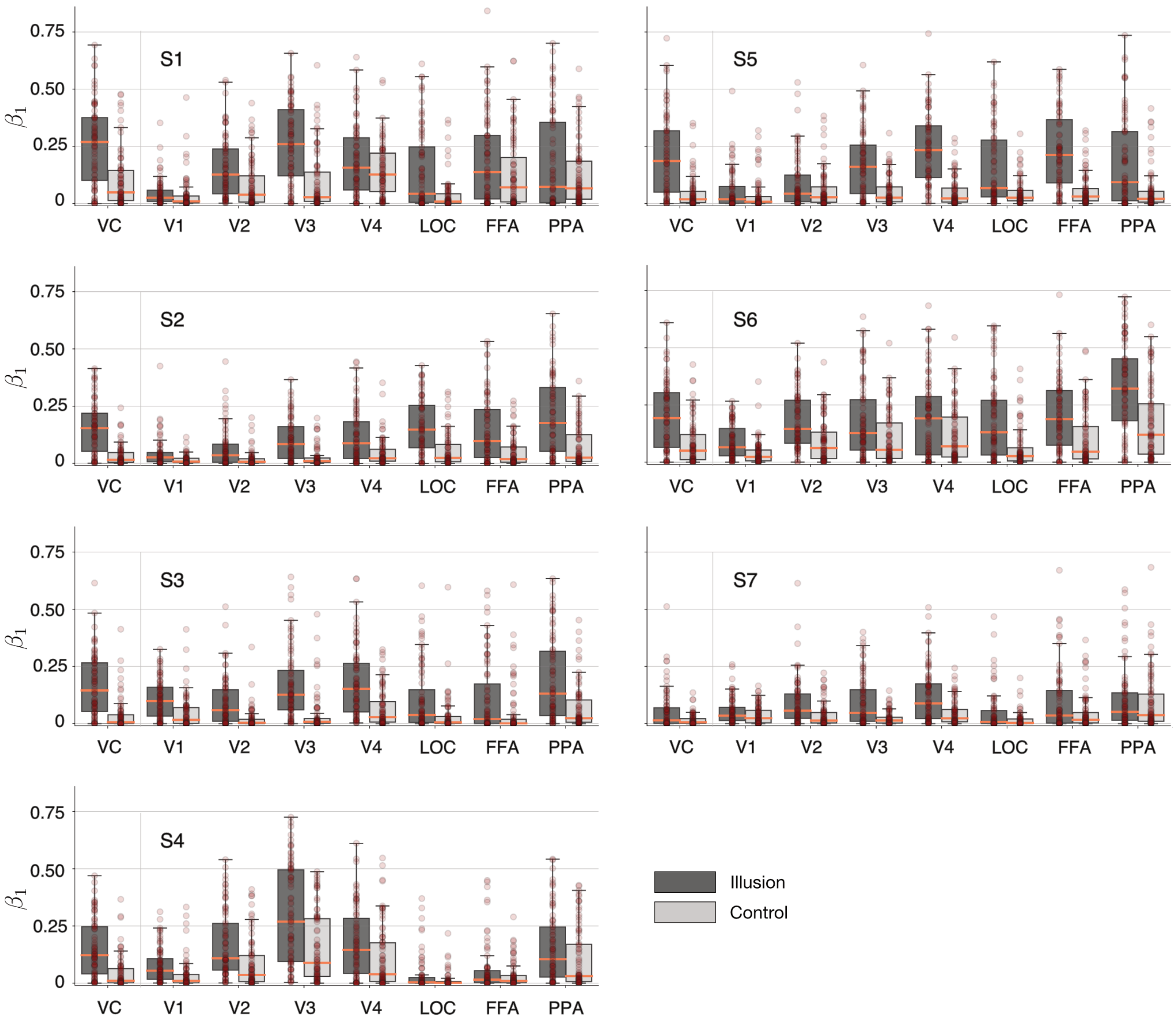

Fig. S20. Comparison of the illusory surface coefficient values between illusion and control conditions of Ehrenstein for individual subjects. Results are based on single-trial reconstructions from VC and individual visual areas. Dots represent individual trials (the degree of opacity provides an indication of the density of dots). Coral lines show the median value and shaded areas of boxplots show the inter-quantile range of trials.

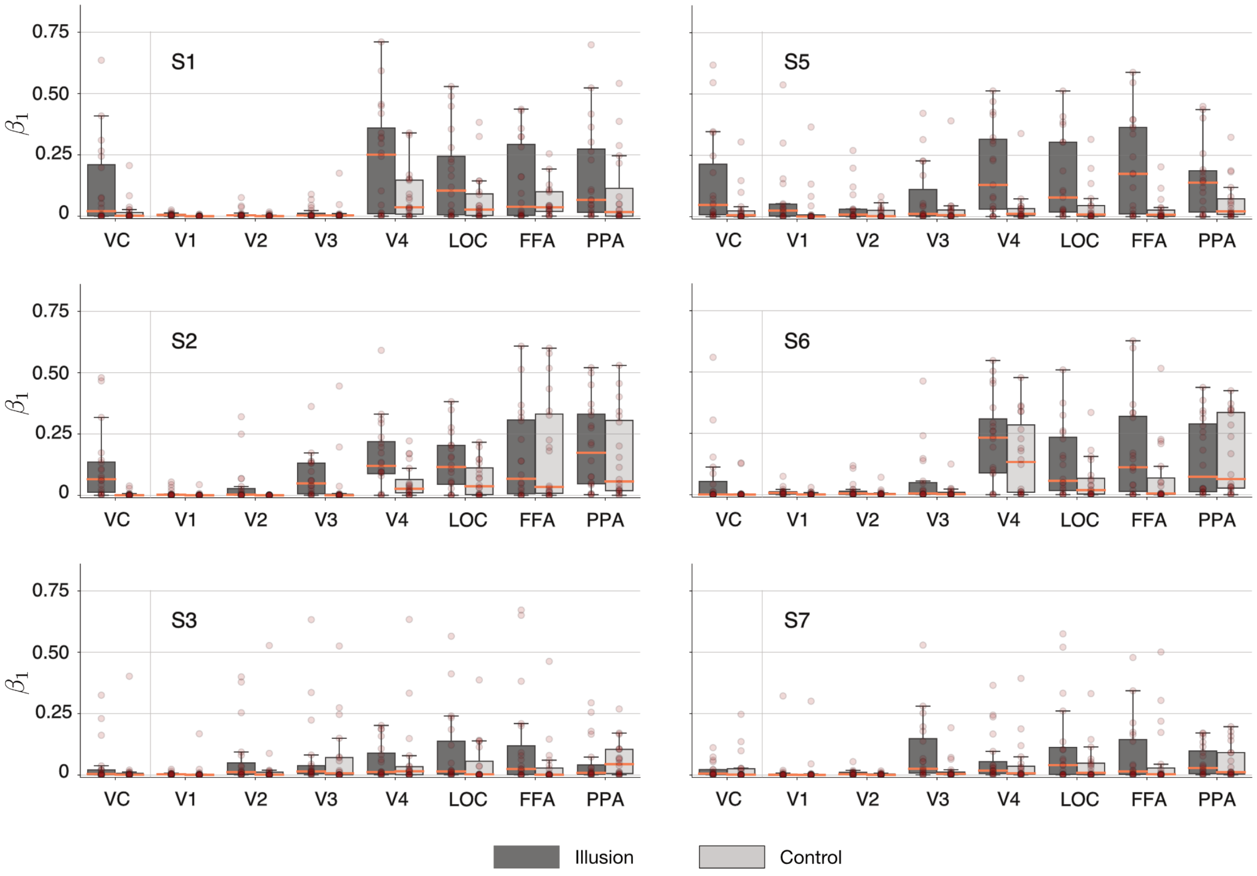

Fig. S21. Comparison of the illusory surface coefficient values between illusion and control conditions of Varin for individual subjects. Results are based on single-trial reconstructions from VC and individual visual areas. Dots represent individual trials (the degree of opacity provides an indication of the density of dots). Coral lines show the median value and shaded areas of boxplots show the inter-quantile range of trials.

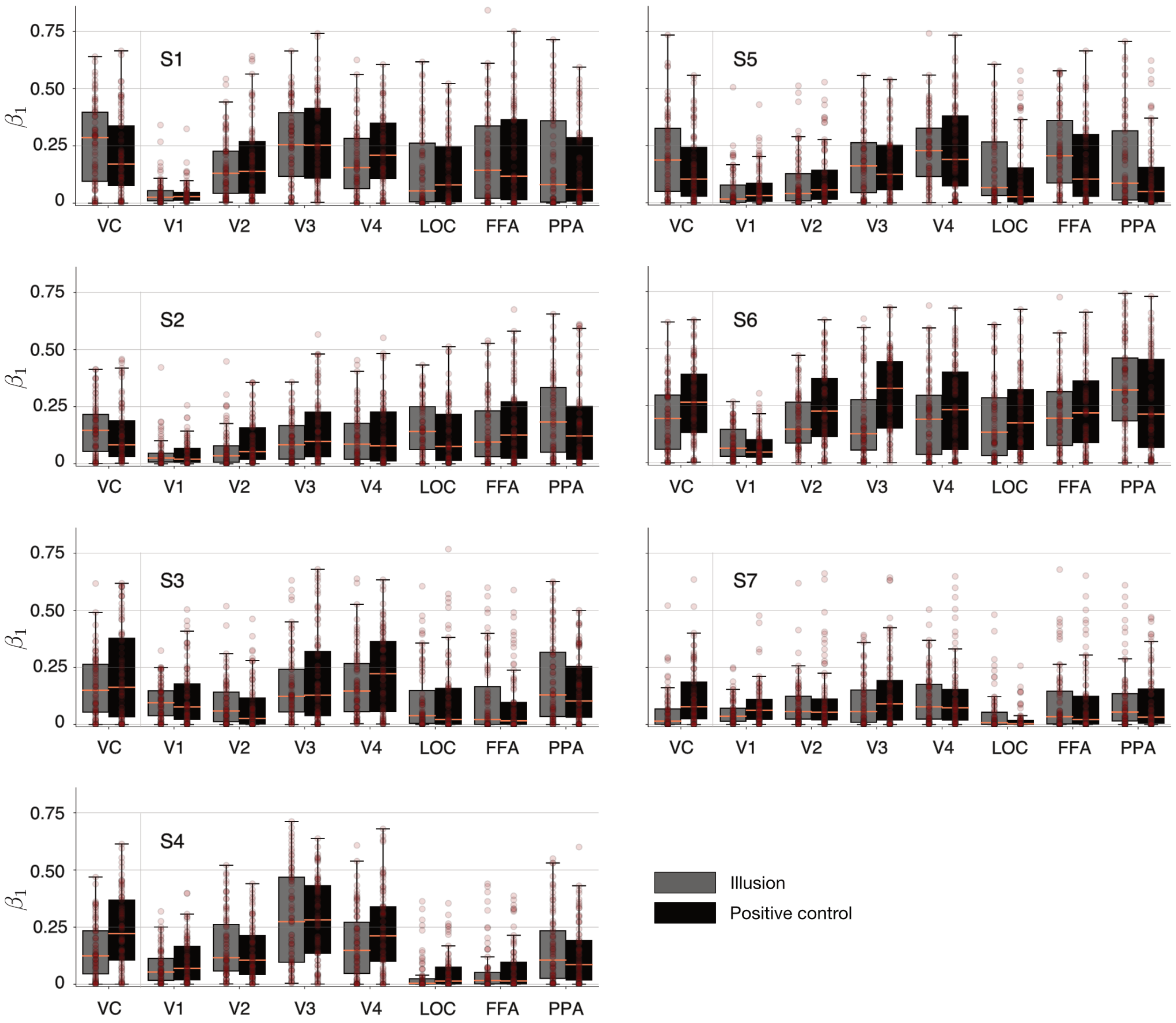

Fig. S22. Comparison of the illusory surface coefficient values between illusion and positive control conditions of Ehrenstein for individual subjects. Results are based on single-trial reconstructions from VC and individual visual areas. Dots represent individual trials (the degree of opacity provides an indication of the density of dots). Coral lines show the median value and shaded areas of boxplots show the inter-quantile range of trials.

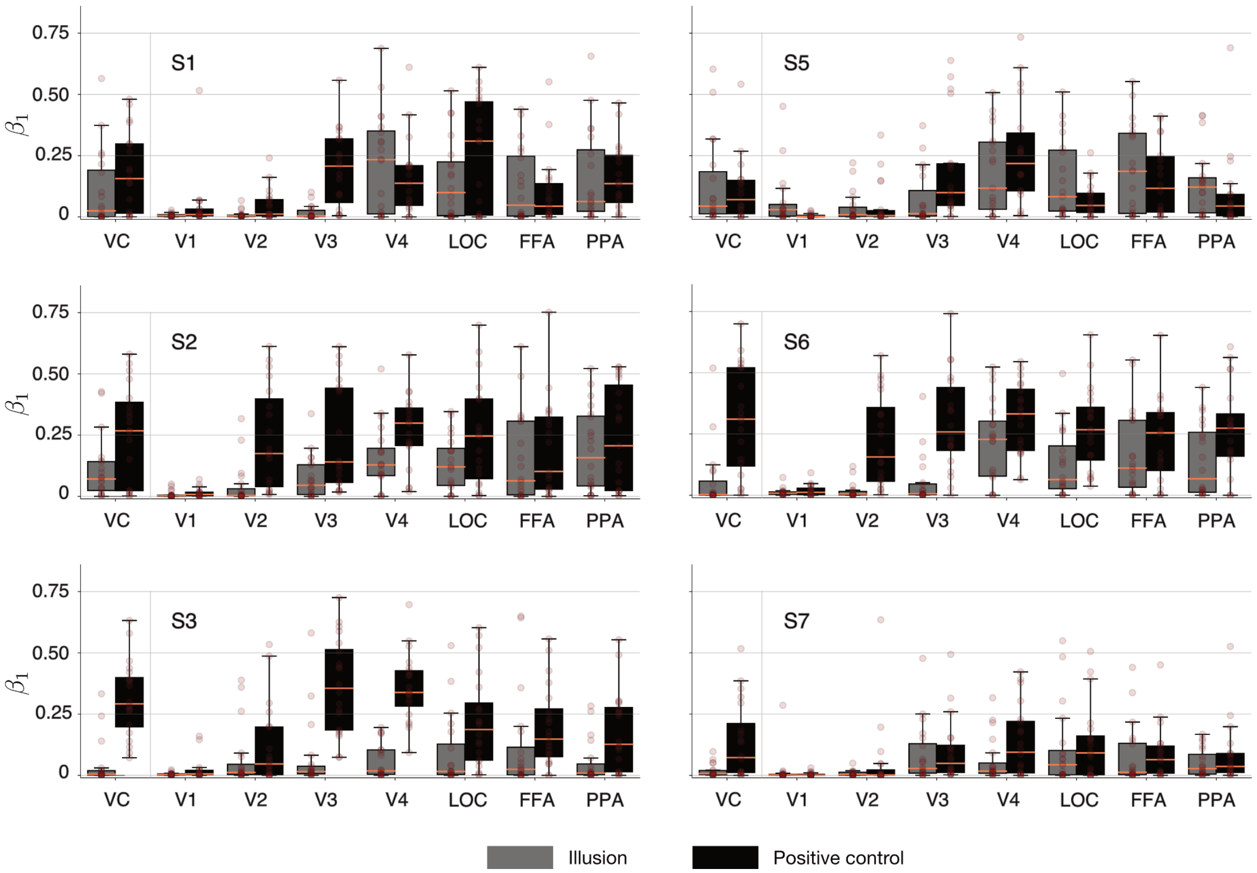

Fig. S23. Comparison of the illusory surface coefficient values between illusion and positive control conditions of Varin for individual subjects. Results are based on single-trial reconstructions from VC and individual visual areas. Dots represent individual trials (the degree of opacity provides an indication of the density of dots). Coral lines show the median value and shaded areas of boxplots show the inter-quantile range of trials.

Movie S1. Reconstructions of illusory and control images using GAN-based generator. A pair of stimulus images (top and bottom) are shown for comparison (left, presented images; middle and right, reconstructed images from subject S1 and S2).

Movie S2. Reconstructions of illusory and control images using diffusion method. The diffusion process is shown. A pair of stimulus images (top and bottom) are shown for comparison (left, presented images; middle and right, reconstructed images from subject S1 and S2).

Movie S3. Reconstructions of illusory and control images using pixel optimization (iCNN). The iterative optimization process is shown. A pair of stimulus images (top and bottom) are shown for comparison (left, presented images; middle and right, reconstructed images from subject S1 and S2).
